# Synthesis of the Brazilian Poduromorpha (Collembola: Hexapoda) with Special Emphasis on the Equatorial Oceanic Islands

**DOI:** 10.3390/insects12030268

**Published:** 2021-03-22

**Authors:** Estevam C. A. de Lima, Maria Cleide de Mendonça, Gabriel Costa Queiroz, Tatiana Cristina da Silveira, Douglas Zeppelini

**Affiliations:** 1 Laboratório de Apterygotologia, Departamento de Entomologia, Museu Nacional, Universidade Federal do Rio de Janeiro, Rio de Janeiro 20940-040, Brazil; cleidecollembola@gmail.com (M.C.d.M.); gabriel_cq@yahoo.com.br (G.C.Q.); tatisilveira22@gmail.com (T.C.d.S.); 2 Laboratório de Sistemática de Collembola e Conservação—Coleção de Referência de Fauna de Solo—CCBSA—Universidade Estadual da Paraíba Campus V, João Pessoa 58070-450, Brazil; zeppelini@daad-alumni.de

**Keywords:** insular fauna, new species, distribution, Collembola

## Abstract

**Simple Summary:**

Endemic Collembola species are bioindicators of environmental quality since native species abundance is particularly sensitive to environmental disturbances. Oceanic island biota generally present high percentages of endemic species, and the vulnerability of these species is higher than those of the continents. The objective of this work was to carry out a survey of the Collembola species of the order Poduromorpha in the Brazilian oceanic islands and synthesize a distribution list of this order for Brazil. Our results reveal four new species of Collembola Poduromorpha for Brazilian oceanic islands that may be useful for the conservation strategies of these island regions and a contributor to the knowledge of the order in Brazil.

**Abstract:**

We present new species and records of Poduromorpha for the Brazilian oceanic islands and synthesis of this order in Brazil. *Friesea noronhaensis* sp. nov., *Friesea rochedoensis* sp. nov., *Willemia insularum* sp. nov. and *Paraxenylla zeliae* sp. nov. are described and a diagnosis of the morphospecies *Acherontiella* sp. Lima and Zeppelini 2015 is provided. We present comparative tables, distribution and taxonomic keys of the *Friesea*, *Arlesia*, *Brachystomella*, *Acherontiella*, *Paraxenylla*, *Xenylla*, and *Willemia* found in the Brazilian oceanic islands and their respective congeners recorded in Brazil.

## 1. Introduction

The order Poduromorpha Börner, 1913 is represented by Collembola with an elongated body, thorax with three separate segments and prothorax with chaetae. Members of this order, about 3400 species, occur in all zoogeographic regions [1].

Abrantes et al. [2] summarized a list of Brazilian Collembola with 102 species of Poduromorpha and 2 morphospecies (*Mesogastrura* cf. *ojcoviensis* and *Mesaphorura* sp. gr. *atlantica*), none of them recorded in Brazilian oceanic islands.

Oceanic islands generally have a high percentage of endemic species. However, in Brazil, the only survey of the Collembola fauna in oceanic islands was done by Lima and Zeppelini in 2015 [3], who presented 36 new records of Collembola, among them 4 species and 3 morphospecies of the Poduromorpha order: *Friesea cubensis* Potapov and Banasko, 1985; *Brachystomella agrosa* Wray, 1953; *Xenylla yucatana* Mills, 1938; *Isotogastrura mucrospatulata* Palacios-Vargas, Lima and Zeppelini, 2013; *Acherontiella* sp.; *Arlesia* sp. and *Willemia* sp., all from Fernando de Noronha Archipelago.

We present results of recent surveys of the Archipelago of São Pedro e São Paulo, Atol das Rocas (Rocas Atoll) and the taxonomic advances for Fernando de Noronha Archipelago after Lima and Zeppelini [3]. *Friesea noronhaensis* sp. nov., *Friesea rochedoensis* sp. nov., *Willemia insularum* sp. nov. and *Paraxenylla zeliae* sp. nov are described. We provide comparative tables, distribution, remarks and taxonomic keys to Brazilian species of the genera *Friesea*, *Arlesia*, *Brachystomella*, *Acherontiela*, *Paraxenylla*, *Xenylla*, *Willemia*, *Isotogastrura,* as well as an update of the distribution and habitat synthesis of the Brazilian Poduromorpha, including contributions after Abrantes et al. [2].

## 2. Materials and Methods

### 2.1. Poduromorpha Survey of the Brazilian Oceanic Islands

**Figure 1:**
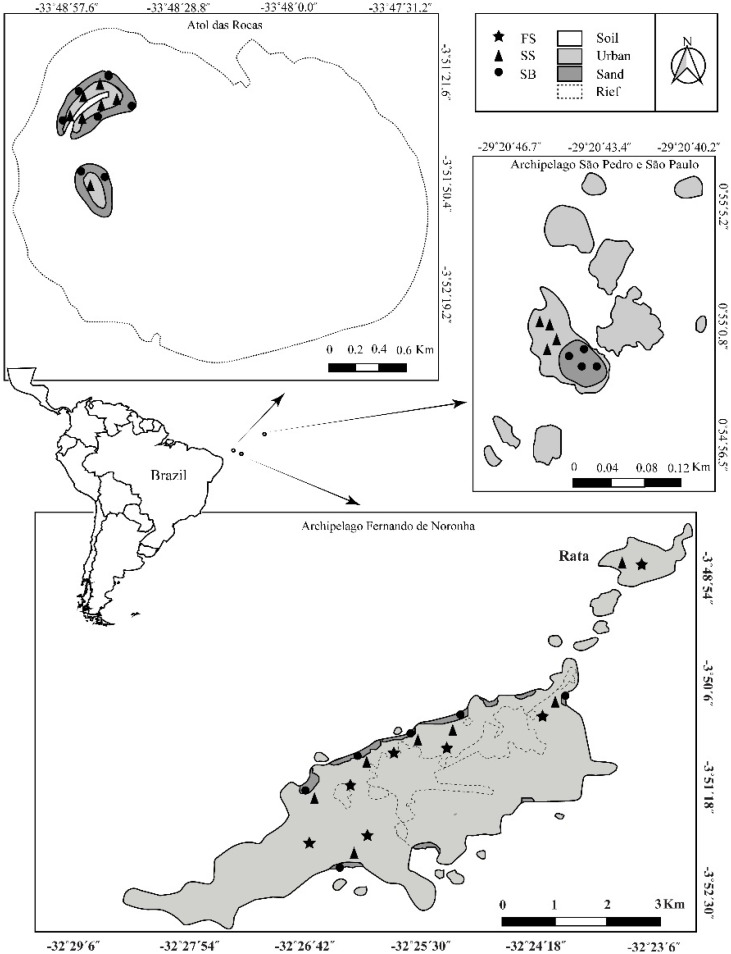
Brazilian oceanic island of the Equatorial Atlantic Ocean. Map show distribution of sites sampled in Fernando de Noronha archipelago, Atol das Rocas (Rocas Atoll) and São Pedro e São Paulo Archipelago.

This survey was conducted in 3 Brazilian oceanic islands (2 Archipelagos and 1 Atoll) located in the Equatorial Atlantic Ocean, all belonging to the Brazilian national territory, as follows:

Fernando de Noronha Archipelago—AFN (3°50′ S, 32°15′ O), São Pedro e São Paulo Archipelago—ASPSP (0°55′ N, 29°20′ O) and Atol das Rocas (Rocas Atoll)—AT (3°51′ S, 33°48′ O) about 360, 1010 and 267 km from the American continent, respectively (Figure 1).

### 2.2. Sample Procedure

The specimens were collected in three coastal environmental areas: the intertidal zone in sand or rock areas, without vegetation, referred to as sandy beach (SB); the sloping land closest to the intertidal zone, with vegetation, referred to as slope forest (SF) and the forest more distant from the beach, referred to as top forest (TF), this latest area only exists in Fernando de Noronha.

Specimens were preserved in ethanol, cleared in Nesbitt’s fluid and then mounted on glass slides in Hoyer’s medium [4]. Figures were elaborated in computer graphic software, photographs and measurements were obtained using a Zeiss Axio Scope A1 microscope with Axiocam 105 color, and Zen 2 Blue software, and deposited in the Coleção de Referência de Fauna de Solo at the Universidade Estadual da Paraíba, João Pessoa, PB, Brazil.

### 2.3. Taxonomic Procedure

The nomenclature of furcal development stages follows Cassagnau 1958. Antennal S-chaetae are numbered from S1 to S8 [5]. Dorsal chaetotaxy nomenclature follows the chaetal group system [6,7,8].

### 2.4. Species List of Brazilian Poduromorpha

Species records, collection location, habitat and biotope information for each species were modified from [2], included: newly described species, new records published between 2012 and 2019 and a survey of the Brazilian oceanic islands between 2012 and 2017. Publications that do not provide identifications to continental species were omitted except genus and morpho-species from Brazilian oceanic islands. Information on the world distribution of species was based on [9] and original new records. Biogeographical distribution regions according to [10], modified by [9,11] as follows: Boreal (Bor) include regions 1–8, Neotropical (Neo) regions 24–30, South African (SAf) region 31, Paleotropical (Pal) regions 9–23, Australian (Aus) regions 32–34, and Antarctic (Ant) regions 35–37. Species restricted to Brazil were based on Culik and Zeppelini [9], defining the Brazilian biogeographic subregions: Amazon (Amz), North and Central Brazil (NCB) and Pampa (Pam) corresponding to biogeographic regions 26, 27 and 29, respectively [9]. Species distributed, at least, in four of the major regions (Neo, Pal, etc.) are considered cosmopolitan (Cos).

### 2.5. Abbreviations

Abd—abdominal segment, Ant—antennal segment, Cd—cephalic diagonal, PAO—postantennal organ, Oc—ocular, ta—anterior trichobothria, Th—thoracic segment, Tita—tibiotarsus, tm—medial trichobothria, tl—lateral trichobothria, tp—posterior trichobothria, Di—dorsointernal group of chaetae, De—dorsoexternal group of chaetae, DL—dorsolateral group of chaetae, CRFS-UEPB—Coleção de Referência da Fauna de Solo-Universidade Estadual da Paraíba, IUCN—International Union for Conservation of Nature. Brazilian states abbreviations according to Table 1.

## 3. Results

The Brazilian Poduromorpha fauna is recorded in the following states and oceanic islands (Table 1).

### 3.1. Poduromorpha Survey of the Brazilian Equatorial Oceanic Islands

The Poduromorpha fauna of the Brazilian oceanic islands is represented by 9 species, as follows:

#### 3.1.1. Neanuridae Börner, 1901

##### *Friesea noronhaensis* sp. nov. Lima and Zeppelini

*Type material.* Holotype male on the slide, Brazil, Fernando de Noronha Archipelago, Rata Island, (3°48′45.61″ S; 32°23′26.17″ O), SF, 19.vii.2012, E.C.A. Lima and A.S. Ferreira leg., deposited at the CRFS-UEPB # 14574. Paratypes: 2 females and 2 males on slides, same data as holotype, deposited at the CRFS-UEPB # 14572, 14573, 15060, 1563.

*Description.* Habitus typical of the genus. Color in ethanol dark gray. Body with secondary integuments. Body length average: 512 µm (*n* = 5); holotype measurements in Table 2.

**Figure 2:**
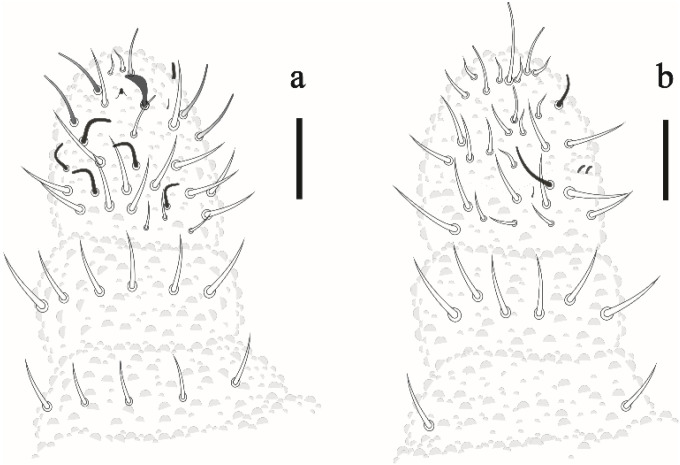
*Friesea noronhaensis* sp. nov. (**a**) dorsal view of antenna (**b**) ventral view of antenna. Scale bar: 10 μm.

Antenna. Antenna shorter than cephalic diagonal (Table 2). Ant IV with 6 S-chaetae (S1–4, S7–8), 1 small ventrolateral S-microchaeta and 1 small subapical organite. Apical bulb simple; with 5 dorsal clavate chaetae and about 12 smooth chaetae ventrally. Sensory organ of Ant III consisting of 2 small subcylindrical, bent, internal S-chaetae covered by a fold of the integument, 2 subcylindrical guard S-chaetae Sgd and Sgv, and 1 ventral S-microchaeta. Ant II with 12 chaetae; Ant I with 7 chaetae (Figure 2a,b).

**Figure 3:**
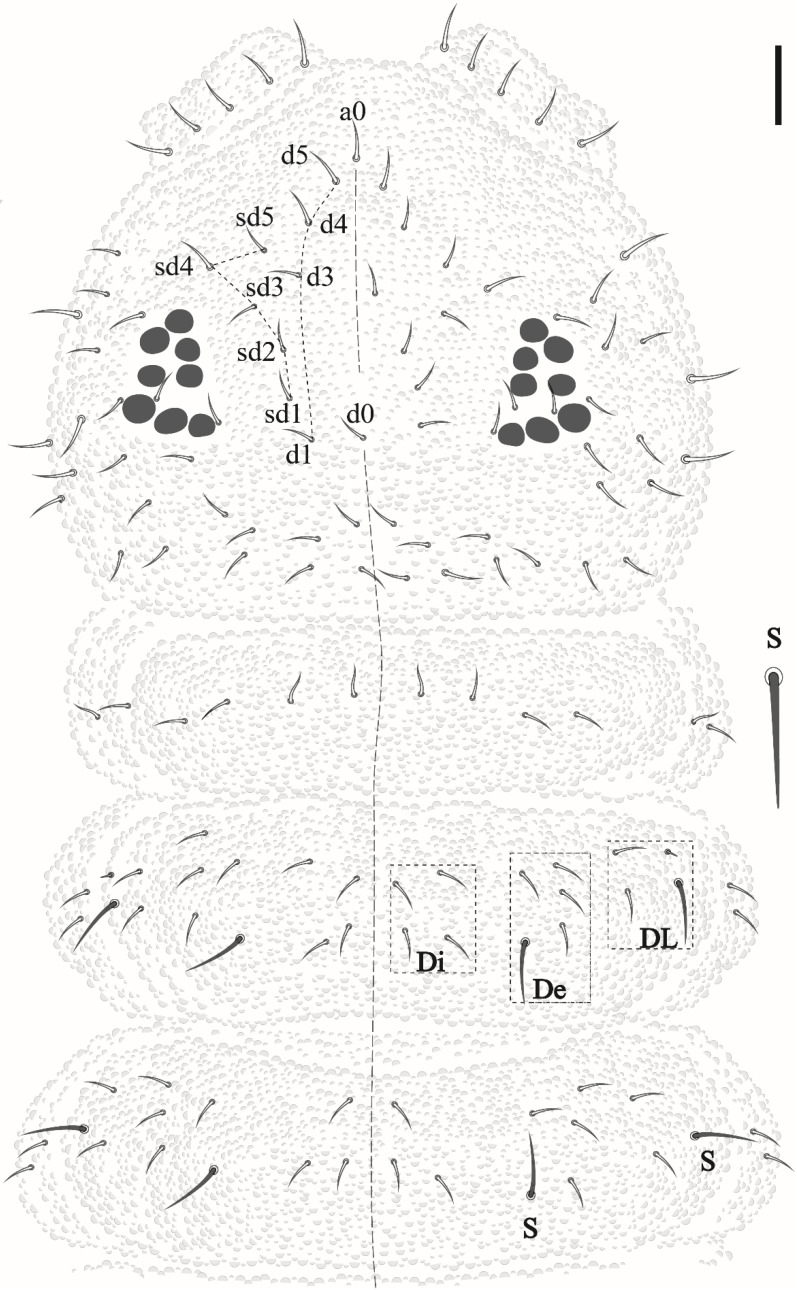
*Friesea noronhaensis* sp. nov, dorsal view of head and Th I–III. Scale bar 10 μm.

**Figure 4:**
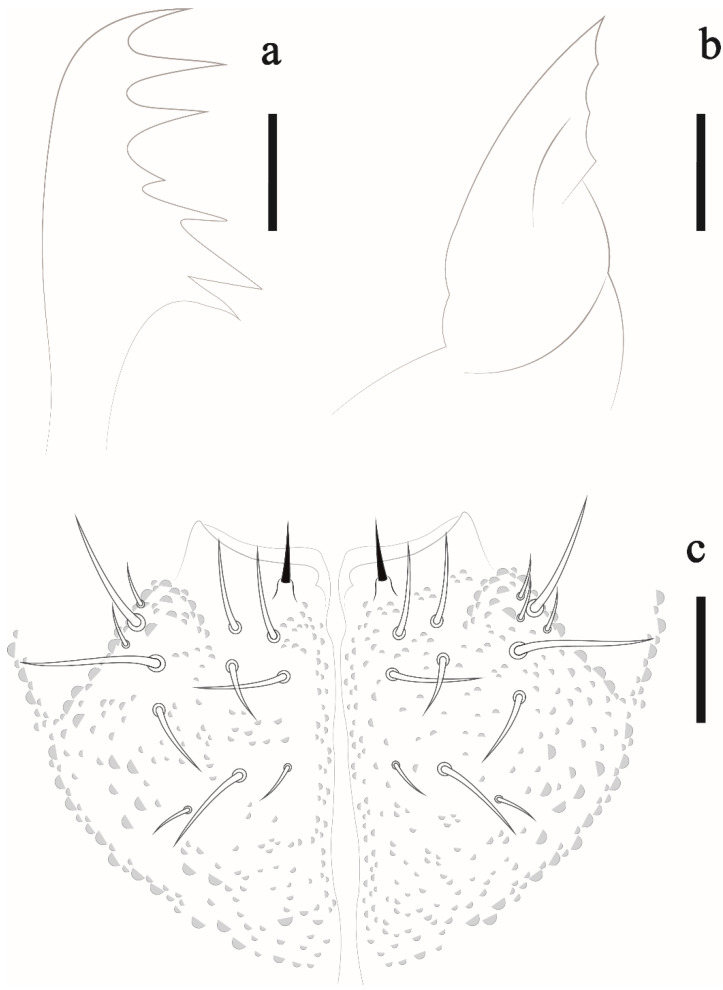
*Friesea noronhaensis* sp. nov. (**a**) mandible, scale bar: 5 μm. (**b**) maxilla, scale bar: 5 μm. (**c**) labium, scale bar: 10 μm.

Head. Eyes 8 + 8 in dark ocular plate; PAO absent; chaetae a0, d0 and 3 Oc present (Figure 3). Pre-labral/labral formula: 4/3,3,4. Mandible with seven teeth, three apical, a medial and three basal (Figure 4a). Maxilla typical of the genus (Figure 4b). Labium with papillated chaeta L (Figure 4c).

**Figure 5:**
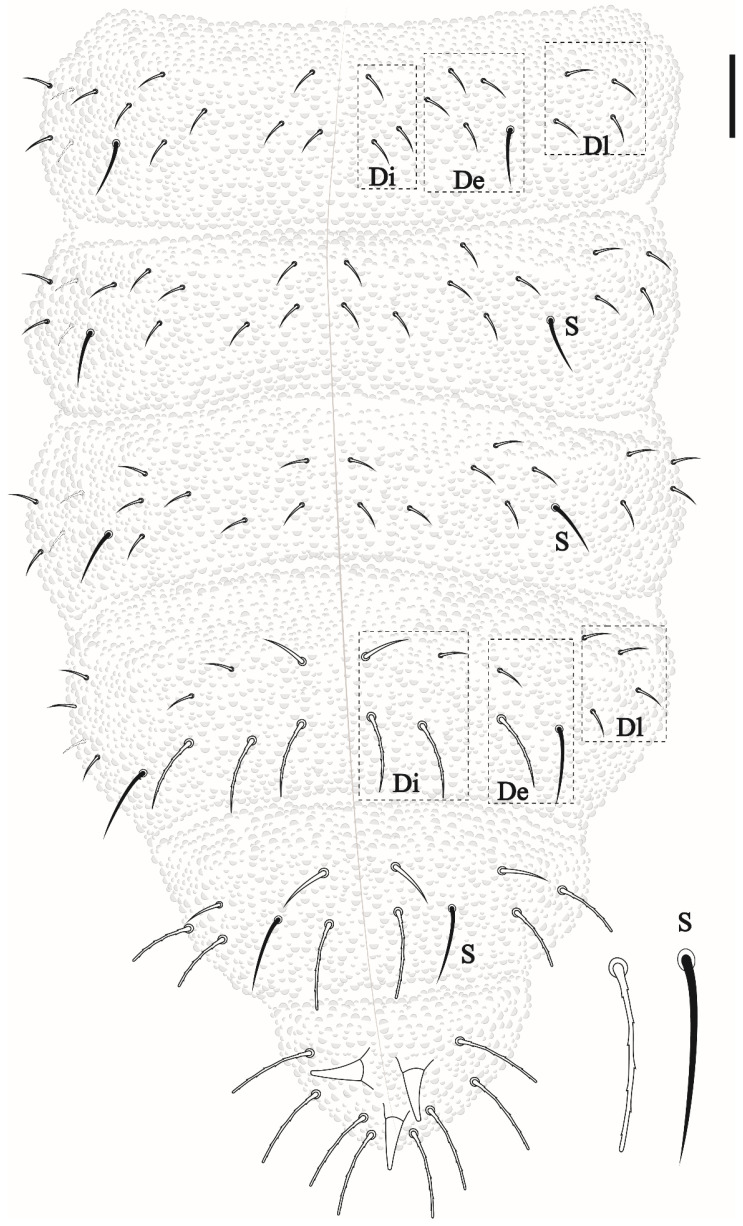
*Friesea noronhaensis* sp. nov. 8, dorsal view of abdominal segments I–VI. Scale bar 10 μm.

Head and body chaetotaxy composed of slightly rugged acuminate ordinary chaetae. S-chaetae smooth, except on Th II–III in DL that are roughly blunt. S-chaetae measuring about twice the size of anterior ordinary chaetae in Th II–III and Abd I–IV. Abd V–VI with posterior strong rough, blunt chaetae longer than others of the body. Sensillar formula by half tergum: 022/11111 (Figure 3 and Figure 5).

Thoracic chaetotaxy. Th I: 4 + 4; Th II: 13 + 13 (Di: 4 chaetae; De: 4 chaetae + 1 S-chaetae; Dl: 2 chateae, 1 S-chaetae +1 ms); Th III: 11 + 11 (Di: 3 chaetae; De: 4 chaetae +1 S-chaetae; Dl: 2 chaetae +1 S-chaetae) see Figure 3.

Abdominal chaetotaxy. Abd I–III: 12 + 12 (Di: 3; De: 4 + 1 S-chaetae; Dl: 4). Abd IV: 11 + 11 (Di: 4; De: 2 + 1 S-chaetae; Dl: 4). Abd V: 6 + 6 (3 anterior acuminate chaetae and 3 posterior blunt chaetae measuring about twice the size than anterior plus 1 + 1 S-chaetae in p2) chaetae. Abd VI with 8 clavate chaetae and 3 anal spines (Figure 5), 2 in a1 position and the third as MO.

**Figure 6:**
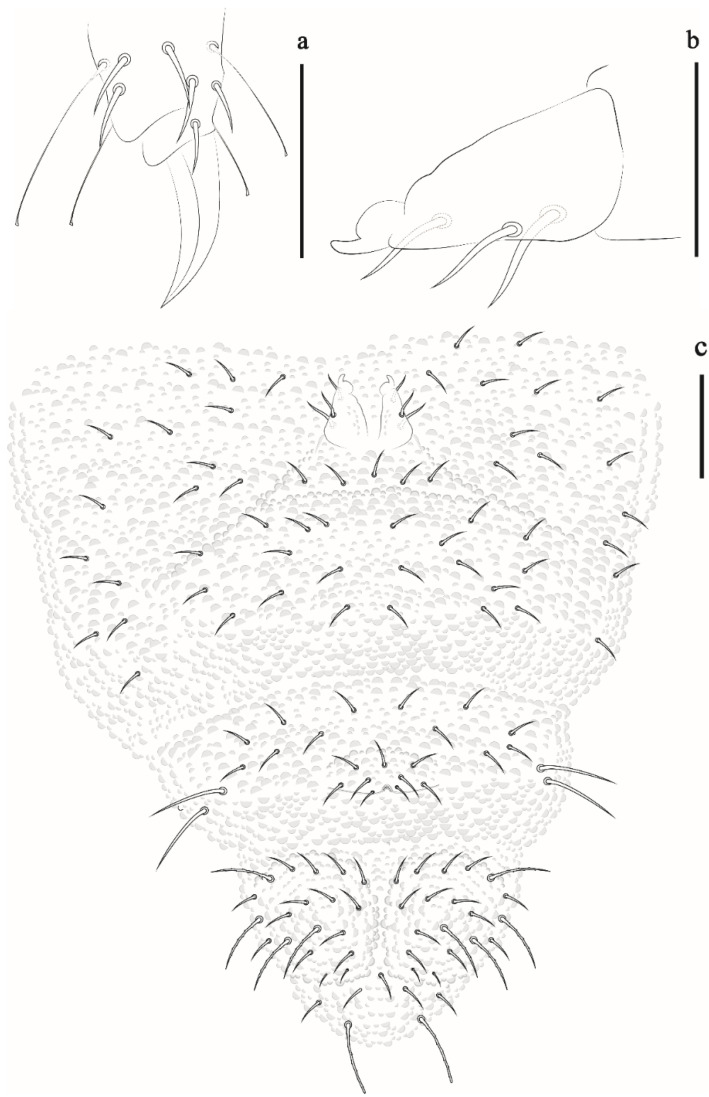
*Friesea noronhaensis* sp. nov. (**a**) Tita III, scale bar: 10 μm. (**b**) furca, scale bar: 10 μm. (**c**) ventral view of abdominal segment II–VI, scale bar: 20 μm.

Appendages. Tita I–III: 18, 18, 17 chateae, with 3, 4, 4 longer and clavate tenent hair, respectively (Figure 6a); Femora I–III: 10, 14, 14 chaetae; trochanters I–III: 5, 5, 5 chaetae; coxae I–III: 3, 7, 7 chaetae; subcoxae I–III with 0, 2, 2 chaetae; epicoxae I–III with 1, 2, 2. Unguis without teeth. Ventral tube with 4 + 4 chaetae. Tenaculum with 2 + 2 teeth and furca state 1 (Figure 6b). Genital plate of female and male with 9 and 28 chaetae, respectively. Anal valves with 16 + 16 chaetae, each anal valve with 2 hr chaetae (Figure 6c).

*Etymology*. Species name derives from the type locality, popularly known as Noronha.

Remarks. *Friesea noronhaensis* sp. nov. is similar to *F. cubensis* Potapov and Banasco, 1985; *F. furculata* Deharveng and Bedos, 1991; *F. rubeni* Deharveng and Bedos 1991 and *F. australica* Greenslade and Deharveng, 1997 by developed furca, dens with 3 chaetae and separate mucro. *Friesea cubensis* and the new species presents 3 anal spines, while *F. furculata*, *F. rubeni* and *F. australica* feature 0, 2 and 4, respectively. The new species differs from *F. cubensis* by posterior chaetae blunt versus acuminate in Abd V–VI and tenent hair clavate versus acuminate. For morphological comparisons between *Friesea* species recorded in Brazil, see Table 3.

*Distribution*. *Friesea noronhaensis* sp. nov. is registered only in the Fernando de Noronha Archipelago on Fernando de Noronha Island (SF sites) and Rata Island (SF and TF sites). The specimens were collected in superficial soil and leaf.

##### *Friesea rochedoensis* sp. nov. Lima and Zeppelini

*Type material*. Holotype female on slide, Brazil, São Pedro e São Paulo Archipelago, Belmonte Island, (0°54′59.98″ N; 29°20′44.03″ O), guano over rocks, May 2017, E.C.A. Lima and D. Zeppelini leg., deposited at the CRFS-UEPB # 14571. Paratypes: 2 males on slides, Brazil, São Pedro e São Paulo Archipelago, Belmonte Island, (0°54′59.74″ N; 29°20′44.57″ O), guano over rocks, 24 April 2017, E.C.A. Lima and D. Zeppelini leg., deposited at the CRFS-UEPB # 14569, 14570. 

*Description.* Habitus typical of the genus. Light blue color in ethanol. Body with well-developed secondary integuments. Body length average: 995 µm (*n* = 5); holotype measurements as in Table 2.

**Figure 7:**
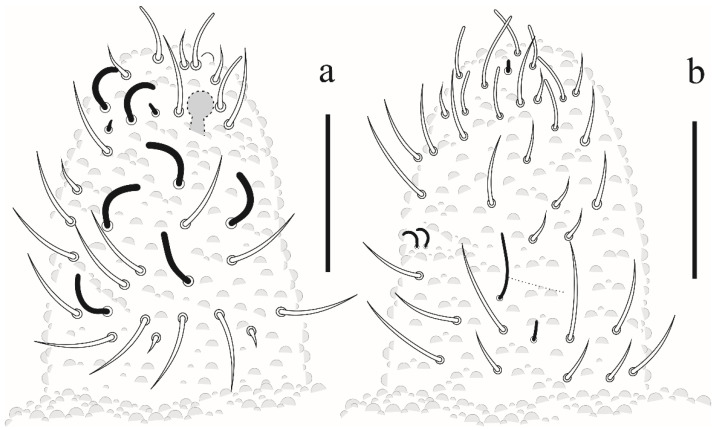
*Friesea rochedoensis* sp. nov. (**a**) dorsal view of antenna, scale bar: 20 μm. (**b**) ventral view of antenna, scale bar: 20 μm.

Antenna. Antenna shorter than cephalic diagonal (Table 2). Ant IV dorsally with 6 S-chaetae (S1–4, S7–8), 1 small ventrolateral S-microchaeta and 1 small subapical organite; apical bulb simple ventrally; with 3–4 dorsoapical and 10–12 clavate plus 5 smooth ventroapical chaetae. Sensory organ of Ant III consisting of 2 small subcylindrical, bent, internal S-chaetae covered by a fold of the integument, 2 subcylindrical guard S-chaetae Sgd and Sgv, and 1 ventral S-microchaeta. (Figure 7a,b). Ant I with 7 chaetae; Ant II with 12 chaetae.

**Figure 8:**
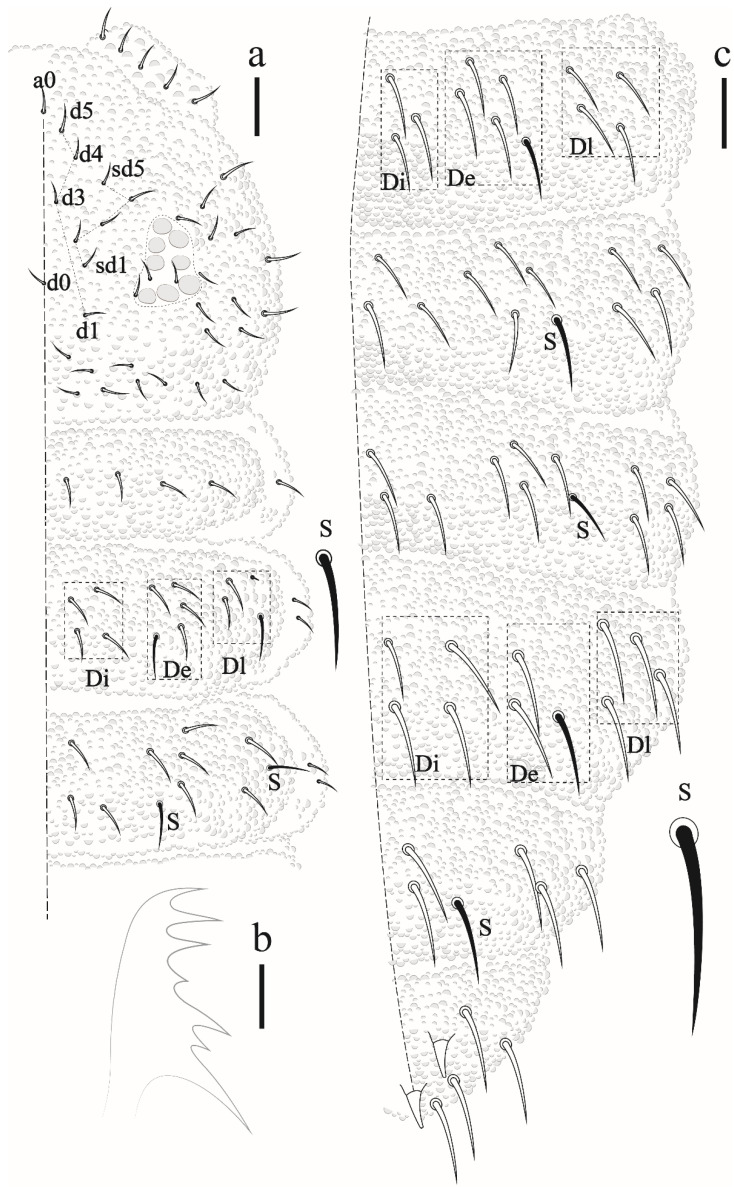
*Friesea rochedoensis* sp. nov. (**a**) dorsal view of head and Th I–III, scale bar: 20 μm. (**b**) mandible, scale bar: 20 μm. (**c**) dorsal view of abdominal segments I–VI, scale bar: 20 μm.

Head (Figure 8a). Eyes 8 + 8 in dark ocular plate; PAO absent; chaetae a0, d0 and 3 Oc present. Pre-labral/labral formula: 4/3,3,4. Mandible with seven teeth, three apical, a medial and three basal (Figure 8b). Maxilla typical of the genus. Labium with papillated chaeta L.

Head and body chaetotaxy composed of slightly rough acuminate ordinary chaetae and S-chaetae about 1/3 longer than ordinary chaetae in Th I–II. Abd I–V S-chaetae subequal or slightly longer than ordinary posterior chaetae. Sensillar formula by half tergum: 022/11111. 

Thoracic chaetotaxy (Figure 8a). Th I: 4 + 4; Th II: 13 + 13 (Di: 4 chaetae; De: 4 chaetae + 1 S-chaetae; Dl: 2 chaetae, 1 S-chaetae + 1 ms); Th III: 11 + 11 chaetae (Di: 3 chaetae; De: 4 chaetae +1 S-chaetae; Dl: 2 chaetae +1 S-chaetae).

Abdominal chaetotaxy (Figure 8c). Abd I–IV: 12 + 12 (Di 3; De 5; Dl 4, posterior chaetae about 1/4 longer than size anterior chaetae). Abd V: 6 + 6 (3 anterior chaetae and 3 posterior of which one S-chaetae subequal chaetae). Abd VI with 6 chaetae and 3 anal spines.

**Figure 9:**
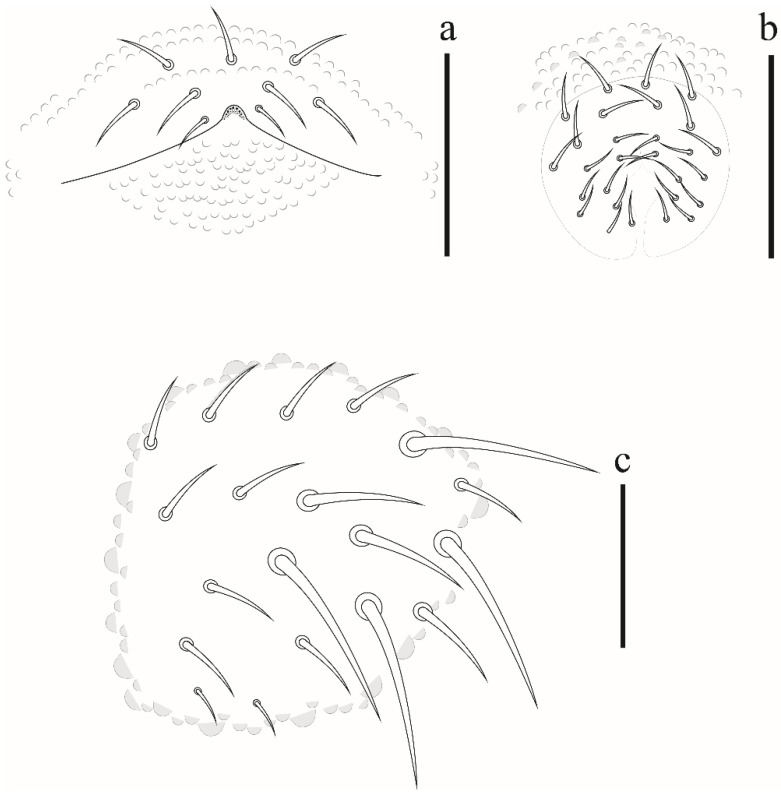
*Friesea rochedoensis* sp. nov. (**a**) female genital plate, scale bar: 20 μm. (**b**) male genital plate, scale bar: 20 μm. (**c**) left anal valve, scale bar: 20 μm.

Appendages. Tita I–III: 18, 18, 17 chaetae, with 3, 4, 4 longer and clavate tenent hairs, respectively; Femora I–III: 10, 11, 10 chaetae; trochanters I–III: 5, 5, 5 chaetae; coxae I–III: 3, 7, 7 chaetae; subcoxae I–III with 0, 2, 2 chaetae; epicoxae I–III: 0, 2, 2. Unguis short, without tooth on inner edge. Ventral tube with 4 + 4 chateae. Tenaculum with 2 + 2 teeth and furca type 1. Genital plate of female (Figure 9a) and male with 17–19 and 26–28 chaetae (Figure 9b). Anal valves with 16 + 16 and 2 hr chaetae (Figure 9c).

*Etymology*. Species name derives from the old name of the type locality that was previously known as “Rochedo de São Pedro e São Paulo”.

**Figure 10:**
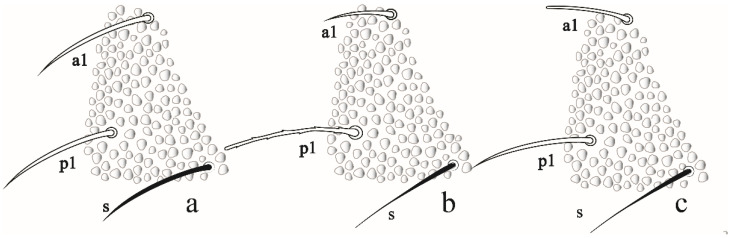
Comparasion between a1, p1 and S-chaetae of abdominal segment V in: (**a**) *Friesea rochedoensis* sp. nov. (**b**) *Friesea noronhaensis* sp. nov. (**c**) *Friesea cubensis* (modified from [6]).

*Remarks*. *F. rochedoensis* sp. nov. is similar *to F. noronhaensis* sp. nov. by chaetotaxy, eyes number, mandible, furca type, number of anal spines and clavate tenent hair. Differs from *F. noronhaensis* by color (light blue vs. dark gray), for absent posterior blunt clavate chaetae in Abd V–VI, by the size (body length 995 vs. 512 µm) and a number of clavate chaetae dorsal and ventroapical of the Ant IV (3–4, 10–12 vs. 5, 0). *Friesea rochedoensis* sp. nov. has hardly differentiated S-chaetae (especially on Abd V) from ordinary chaetae, whereas *F. noronhaensis* sp. nov. and *F. cubensis* S-chaetae are easily differentiated from ordinary chaetae. *Friesea rochedoensis* sp. nov. has chaetae a1 and p1 subequal in Abd V, while *F. noronhaensis* sp. nov and *F. cubensis* have p1 greater than a1 in Abd V (Figure 10a–c). For others, morphological comparisons between *Friesea* species recorded in Brazil, see Table 3.

**Figure 11:**
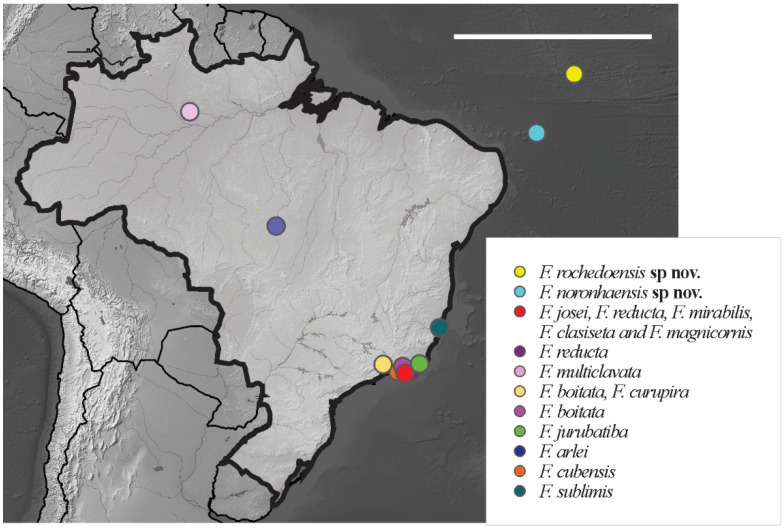
Distribution map for Brazilian species of the genus *Friesea* Dalla Torre, 1895. Scale bar: 3000 km.

*Distribution*. *Friesia rochedoensis* sp. nov. is registered only for Belmont island of the São Pedro e São Paulo Archipelago. The specimens were collected from seabird guano deposits over rocks. For the congeners registered in Brazil, see Figure 11.

##### Identification Key for the Species of *Friesea* Recorded in Brazil

1 Without furca...................................................................................................................... 2

– With furca............................................................................................................................ 7

2 1 + 1 eyes................................................................ *F. curupira* Queiroz and Mendonça, 2015

– With more than 1 + 1 eyes.................................................................................................... 3

3 Modified chaetae of Abd VI are spiniform...................... *F. josei* Palacios-Vargas, 1986

– Modified chaetae of Abd VI are clavate.......................................................................... 4

4 With 10 clavate chaetae on the abd VI............................................................................. 5

– Less than 10 clavate chaetae on Abd VI.......................................................................... 6

5 Th I with 3 + 3 chaetae....................................................................... *F. reducta* Denis, 1931

– Th I with 2 + 2 chaetae....................... *F. multiclavata* Neves, Mendonça and Queiroz, 2019

6 With 6 slightly clavate chaetae on abd VI........... *F. boitata* Queiroz and Mendonça, 2015

– With 8 clavate chaetae on Abd VI................... *F. jurubatiba* Silveira and Mendonça, 2018

7 4 + 4 eyes........................................................................ *F. arlei* Massoud and Bellinger, 1963

– 8 + 8 eyes................................................................................................................................ 8

8 Developed furca (state 1 according to [12])..................................................................... 9

– Reduced furca (state 2 according to [12])........................................................................ 11

9 Modified chaetae of Abd VI clavate and rugged, clavate tenent hair........................................................................................................... *F. noronhaensis* sp. nov.

– Modified chaetae of the Abd VI acuminate................................................................... 10

10 Tenent hair acuminate......................................... *F. cubensis* Potapov and Banasko, 1985

– Tenent hair clavate......................................................................... *F. rochedoensis* sp. nov.

11 With clavate chaetae on Abd VI.................................................................................... 12

– Without clavate chaetae on Abd VI..................................... *F. mirabilis* (Tullberg, 1871)

12 Ant IV with 5 sensilla..................................................................................................... 13

– Ant IV with 6 sensilla................................................................ *F. claviseta* Axelson, 1900

13 Anal spine are the same size............................................. *F. sublimis* Macnamara, 1921

– Posterior anal spine smaller than anterior............................ *F. magnicornis* Denis, 1931

##### *Arlesia albipes* (Folsom, 1927)

*Brazilian Oceanic Island Records*. FN (3°52′8.88″ S; 32°26′13.57″ W), SF, 01 August 2012, E.C.A. Lima, A.S. Ferreira leg., CRFS# 15144–15145.

**Figure 12:**
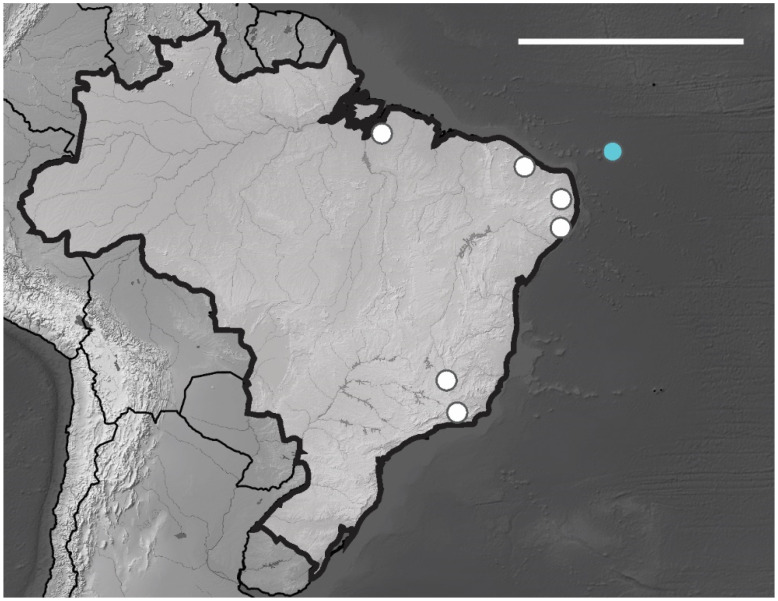
Distribution map for *Arlesia albipes* (Folsom, 1927) records in Brazil. Blue circle, Fernando de Noronha; white circle, Brazilian states. Scale bar: 3000 km.

*Brazilian Occurrence*. In Brazil, this species is widely distributed in the states PA, PE, PB, PI, MG and RJ [13] (Figure 12). In Brazilian oceanic island *Arlesia albipes* recorded only in Fernando de Noronha Archipelago on Fernando de Noronha Island (SF sites). The specimens were collected on superficial soil and leaf litter.

*Biogeographic Distribution*. Type locality of *Arlesia albipes* is Margarita Swamp, Canal Zone, collected by J. Zetek and I. Molino on June 28, 1923, in a termite mound near the base of a tree stump and deposited at U.S.N.M. # 40383. According to [1], this species has been found in the following biogeographic regions: Caribbean mainland (24a), Antillean and South Florida (24b), Venezuela and Guyana (25), Amazon (26), Northeast and Central Brazilian (27) and Andean (28). Neotropical species.

*Remarks.* The specimens found in the Fernando de Noronha Archipelago are in accordance with the original description [14] and updates [15,16]. Lima and Zeppelini [3] recorded as *A*. sp. nov. For comparisons between *Arlesia* species recorded in Brazil, see Table 4.

##### Identification Key for the Species of *Arlesia Handschin*, 1942 Recorded in Brazil

1 Mandible with 4 teeth…………………..……………...…...… *A. albipes* (Folsom, 1927)

– Mandible with more than 4 teeth…………………...……………..……..……..2

2 Black with yellow strips, mandible with 6 teeth……………………………………… ………………………………………………….. *A. arleana* Mendonça and Fernandes, 1999

– Light gray, mandible with 5 teeth…….... *A. intermedia* Fernandes and Mendonça, 2004

#### 3.1.2. Brachystomellidae Stach, 1949

##### Brachystomella agrosa Wray, 1953

*Brazilian Oceanic Island Records*. FN (3°50′54.21″ S; 32°25′45.56″ O), TF, 20 Jul 2012, E.C.A. Lima and D. Zeppelini leg., CRFS# 14400–14401, 14409–14414, 14422. FN (3°50′58.48″ S; 32°26′3.83″ O), SF, 25 July 2012, E.C.A. Lima and A.S. Ferreira leg., CRFS# 14415–14418. FN (3°51′14.32″ S; 32°26′12.62″ O), TF, 25 July 2012, E.C.A. Lima and A.S. Ferreira leg., CRFS# 14450–14455, 14457–14461, 15094–15096, 15098. FN (3°50′18.66″ S; 32°24′3.09″ O), SF, 07 August 2012, E.C.A. Lima and D.D. Silva CRFS# 14456. FN (3°51′3.73″ S; 32°26′0.38″ O), SF, 27 July 2012, E.C.A. Lima and A.S. Ferreira leg., CRFS# 14399, 14419–14421, 14423, 14426–14427. FN (3°50′36.76” S; 32°25′12.64” O), SF, 26 Jul 2012, E.C.A. Lima and A.S. Ferreira leg., CRFS# 9565–9568, 9593, 14449. FN (3°50′48.92” S; 32°25′13.61″ O), TF, 26 July 2012, E.C.A. Lima and A.S. Ferreira leg., CRFS# 9562–9564, 9571–9592. FN (3°48’45.06″ S; 32°23′14.07″ O), TF, 19 Jul 2012, E.C.A. Lima and A.S. Ferreira leg., CRFS# 15063, 15069. FN (3°52′8.88” S; 32°26′13.57″ O), SF, 01 August 2012, E.C.A. Lima and A.S. Ferreira leg., CRFS# 14402–14403, 14405, 14407, 14425, 14429, 14437, 14440. FN (3°52′8.88″ S; 32°26′13.57″ O), SF, 14 Aug 2012, D.D. Silva leg, CRFS# 14408. FN (3°51′50.41″ S; 32°26′5.44″ O), TF, 01 August 2012, E.C.A. Lima and A.S. Ferreira leg., CRFS# 14406. FN (3°51′52.31″ S; 32°26′38.80″ O), TF, 31 July 2012, E.C.A. Lima and A.S. Ferreira leg., CRFS# 14424, 14428, 14435, 14436, 14438–14439, 14443–14444.

**Figure 13:**
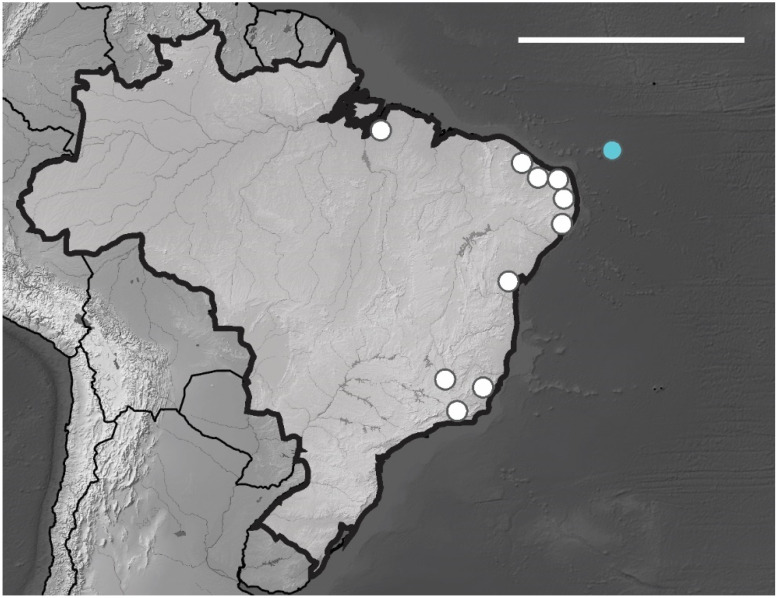
Distribution map for *Brachystomella agrosa* Wray, 1953 recorded in Brazil. Blue circle, Fernando de Noronha; white circles, Brazilian states. Scale bar: 3000 km.

*Brazilian Occurrence*. In Brazilian oceanic island *B. agrosa* recorded in Fernando de Noronha Archipelago on Fernando de Noronha and Rata Island (SF, TF sites), the specimens were collected in superficial soil and leaf litter. In Brazil, this species is widely distributed and has been registered in the following states CE, BA, PI, PE, PB, RN, ES, SP and RJ [13] (Figure 13).

*Biogeographic Distribution*. The type locality of this species is Maricao, Puerto Rico, collected by M. Capriles, take on Road 27, at 120 m altitude. According to [1], this species has been found in the following biogeographic regions: Caribbean mainland (24a), Antillean and South Florida (24b), Venezuela and Guiana (25), Amazon (26), Northeast and Central Brazil (27). Neotropical species.

*Remarks.* In addition to the original description [17], several authors contributed to the description morphological of this taxon, such as: [18,19,20,21]. The specimens of *B. agrosa* examined agree with the above descriptions. For comparisons between *Brachystomella* species recorded in Brazil, see Table 5.

##### Identification Key for the Species of *Brachystomella* Ågren, 1903 Recorded in Brazil

1 2 + 2 eyes……….…………….………………………. *B. garayae* Queiroz and Weiner, 2011

– With more than 2 + 2 eyes………………………….………………………………...….. 2

2 7 + 7 eyes……………………………………………………... *B. septemoculata* Denis, 1931

– 8 + 8 eyes…………………………………………………………………………………… 3

3 With 3 dental chaetae……………………… *B. villalobosi* Cassagnau and Rapoport, 1962

– With more than 3 dental chaetae……………………………………………………….. 4

4 With 5 dental chaetae……………………………………………………………………. 5

– With 6 dental chaetae………………………... *B. ceciliae* Fernandes and Mendonça, 2004

5 Th I with 2 + 2……………………………………………………………………………… 6

– Th I with 3 + 3………………………………………………….. *B. parvula* (Schäffer, 1896)

6 tenent hair acuminate……………………………………...………………..………….. 7

– tenent hair capitated……………………………….. *B. platensis* Najt and Massoud, 1974

7 Apical bulb simple……………………………………………………………………….. 8

– Apical bulb with 3 vesicles……………………………………………………………….. 9

8 PAO with 5 vesicles…………………………..……...…………... *B. contorta* Denis, 1931

– PAO with 4 vesicles………………………….……………………. *B. agrosa* Ågren, 1903

9 PAO with 6 vesicles……………………………….. *B. saladaensis* Weiner and Najt, 2001

– PAO with 4 vesicles………………...…… *B. nordestina* Souza, Bellini and Weiner, 2018

#### 3.1.3. Hypogastruridae Börner, 1906

##### Acherontiella sp.

*Examined material*. Female on slide, Brazil, Fernando de Noronha Archipelago, Fernando de Noronha Island, (3°50′36.76″ S; 32°25′12.64″ O), SF, 26 July 2012, E.C.A. Lima and A.S. Ferreira leg., deposited at the CRFS-UEPB # 14568.

*Diagnosis*. Color in ethanol white. Tegumental granulation rather strong. Body length: 746 µm; measurements as in Table 4.

Ant IV with 5 globular S-chaetae which 2 lateroexternal very large, placed in cavities, and the other 3 are smaller; dorsoexternal microsensillum and small subapical organite; present small non-retractile apical vesicle in ventro subapical position. Ant III and IV fused dorsally, the ventral separation well-marked. Sensory organ of Ant III consisting of two small internal sensilla, two sub-cylindrical (ring-shaped) guard sensilla and ventral microsensillum. Ant II–I with 11 and 7 chaetae, respectively. Eyes and PAO absent. Buccal cone typical of the genus. Pre-labral/labrum formula 4/554 chaetae, anterior labral chaetae spiniform. Dorsal chaetotaxy with rather short, ordinary chaetae, with long thin S-chaetae, their formula per half tergum: 022/11111. Abd VI without anal spines. Ventral tube with 4 + 4 chaetae. Tita I, II and III with 18, 18 and 17 chaetae, respectively. Femora I, II and III with 11, 11,10 chaetae, respectively, trochanters with 3,4,4 chaetae each, coxae I, II and III with 3, 8 and 7 chaetae.

*Remarks*. *Acherontiella* sp. [3] is similar to *Acherontiella candida* (Delamare Deboutteville, 1952), *A. colotlipana* Palacios-Vargas and Thibaud, 1985 and *A. kowalskiorum* Weiner and Najt, 1998 by type of guard sensilla of the Ant III organ (ring-shaped) and number of sensilla in Ant IV (with 5 globular sensilla). *Acherontiella* sp. differs from these species by anterior labrum chaetae (spiniform versus thin and smooth). For comparisons between *Acherontiela* species recorded in Brazil, see Table 6.

**Figure 14:**
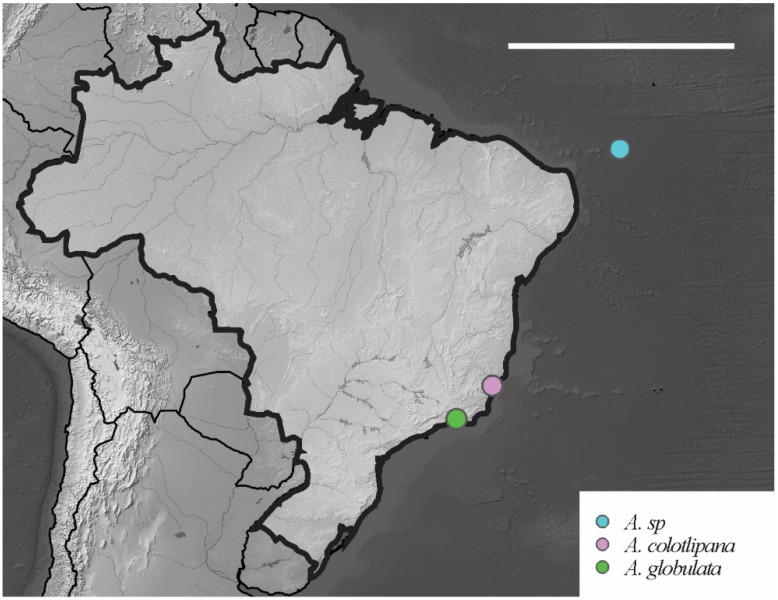
Distribution map for *Acherontiella* Absolon, 1913 species recorded in Brazil. Scale bar: 3000 km.

*Distribution. Acherontiella* sp. was found only on Fernando de Noronha Island in the SF region. The specimen was collected in soil litter. Additional material is required in order to properly describe this potentially new species. For other congeners records in Brazil, see Figure 14.

##### Identification Key for the Species of *Acherontiella* Recorded in Brazil

1 Ant IV with 6 globular sensilla…………….. *A. globulata* Thibaud e Massoud, 1980

- Ant IV with 5 globular sensilla……………….………...……………………………….. 2

2 Labrum with 4 smooth anterior chaetae…………………………………………………. ………………………………………… *A. colotiplana* Palacios-Vargas and Thibaud, 1985

- Labrum with 4 spiniform anterior chaetae…….……. *A.* sp. Lima and Zeppelini, 2015

##### *Paraxenylla zeliae* sp. nov Lima and Zeppelini

*Type material*. Holotype male on slide, Brazil, Atol das Rocas, Cemitério Island, (3°51′47.15″ S; 33°48′54.14″ O), Oct 2015, E.C.A. Lima leg., deposited at the CRFS-UEPB # 14567. Paratypes: 2 females on slides, Brazil, Atol das Rocas, Farol Island, (3°51′28.14″ S; 33°48′44.87″ O), guano, Sept 2015, E.C.A. Lima leg., deposited at the CRFS-UEPB # 14565, 14566.

*Description*. Habitus typical of the genus. Color in ethanol blue. Body length average: 690 µm (*n* = 10); holotype measurements as in Table 2. Body with short acuminate chaetae (7–9 μm). Macrochaetae and S-chaetae (14–18 μm) about twice the length of ordinary chaetae.

**Figure 15:**
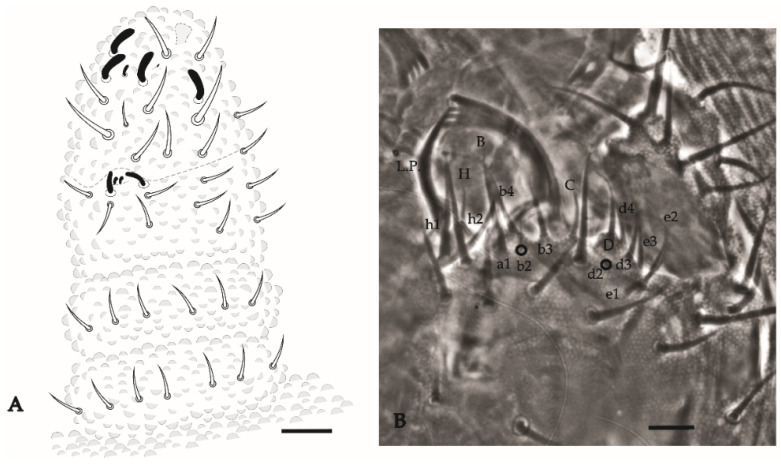
*Paraxenylla zeliae* sp. nov. (**A**), dorsal view of antenna; (**B**), labial palp and mandible apex, nomenclature after [22]. Scale bars: 10 µm.

Antenna (Figure 15A). Ant IV with 4 thick S-chaetae and a small S-microchaeta between the 3 S-chaetae of the external group. Subapical organ present (Figure 15A). Apical bulb simple. Sensory organ of Ant III with 5 S-chaetae (2 small, S3 and S4, behind a fold of the integument, 2 longer guard chaetae S2 and S5, and one external microchaeta S1). Ant II–I with 11 and 7 chaetae, respectively.

Head. Eyes 5 + 5 well pigmented. Labral formula: 27/554 chaetae. Mandible with 4 apical teeth (Figure 15B). Internal process of labium long, labial palps as in Figure 15B. Maxillae with 6 hypertrophied fringed lamellae.

**Figure 16:**
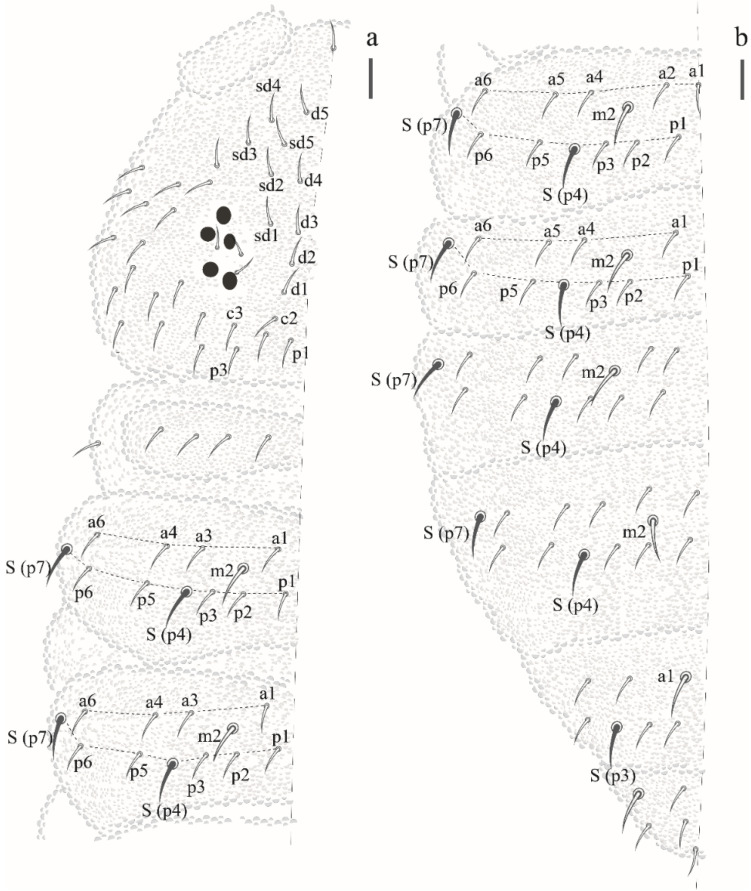
*Paraxenylla zeliae* sp. nov. (**a**) dorsal view of head and Th I–III, scale bar: 10 µm. (**b**) dorsal view of Abd I–VI, scale bar: 10 µm.

Thorax I with 3 + 3 to 4 + 4 dorsal chaetae. Dorsal chaetotaxy of head and Th in Figure 16a; m1 absent, m2 present on Th II–III as macrochaetae, ms on Th II not visualized.

Dorsal chaetotaxy of abdomen as in Figure 16b. Axial S chaetae 44/44,443; m2 on Abd I–III and a1 on Abd V as macrochaetae. Abd VI dorsally with 5 + 5 chaetae, one posterior chaetae and 3 An microchaetae on the supra-anal valve.

**Figure 17:**
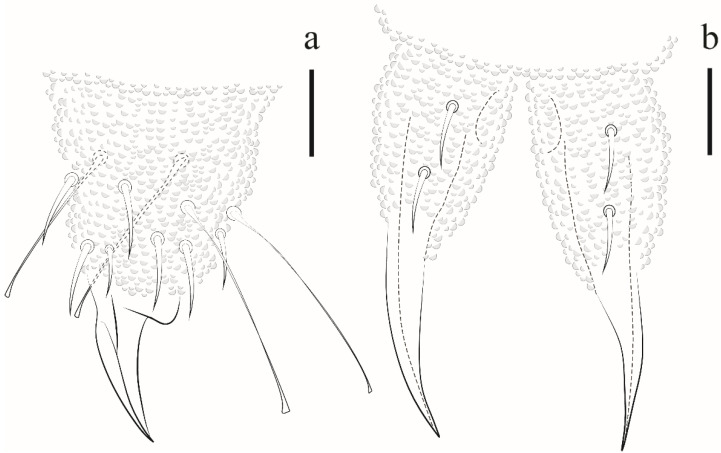
*Paraxenylla zeliae* sp. nov. (**a**) Tita III, scale bar: 20 µm. (**b**) mucrodens, scale bar: 20 µm.

Leg. Subcoxa 1 I–III with 1,3,3 chaetae. Subcoxa 2 I–III with 0,1,1 chaetae. Coxae I–III with 3,6, 7 chaetae. Trochanters with 5 chaetae each, femora I, II, III with 12, 12, 11 chaetae, respectively. On each femur, 1 ventral chaeta longer than others. Tita I, II, III with 19, 19, 18 chaetae, respectively, each with 4 capitate tenent hairs, 2 internal longer than 2 external. Unguis without teeth, no empodial appendix (Figure 17a).

Ventral tube with 1 + 1 chaetae. Abd II with 1 + 1 ventral chaetae, Abd III with 5 + 5 chaetae. Tenaculum with 3 teeth on each ramus. Mucrodens with two chaetae (Figure 17b). Female with 3 + 3 pregenital chaetae, 4–6 circumgenital and 2 eugenital. Male with 3 + 3 pregenital chaetae, 8–10 circumgenital and 4 + 4 eugenital. Each anal valve with 11–13 chaetae. Anus terminal, no anal spines.

*Etymology*. The species is named after Head of the Biological Reserve Atol das Rocas Mrs. Maurizélia de Brito Silva, in honor of a lifetime dedication to conservation and assist scientific research on the Atol das Rocas.

*Remarks*. *Paraxenylla zeliae* sp. nov. is similar to *P. affiniformis* (Stach, 1930) (cosmopolitan), *P. piloua* Thibaud and Weiner, 1997 (New Caledonia), *P. lapazana* Palacios-Vargas and Vázquez, 1988 (Mexico), *P. cubana* Palacios-Vargas and Janssens, 2006 (Cuba), *P. peruensis* Palacios-Vargas and Janssens, 2006 (Peru) *P. mahahualana* Palacios-Vargas and Vázquez, 2018 (Mexico) by ventral tube with 1 + 1 chaetae, Th II–III m1 absent and m2 present, Abd I–III p2 and Abd V a1 as macrochaetae, Abd V S-chaetae in p4 position. The new species differ from *P. cubana* by tenaculum teeth (3 + 3 versus 2 + 2) and capitate tenent hair on Tita III (4 versus acuminate). *Paraxenylla zeliae* sp. nov. has 4,4,4 clavate tenent hair while *P. lapazana* 3,3,3 clavate or acuminate, *P. affiniformis* 5,5,5 acuminate and *P. mahahualana* 5,5,5 clavate. The new species share the same number of tenent hair with *P. piloua* and *P. peruensis*. However, *P. zeliae* sp. nov. differ from *P. piloua* by tenent hair morphology (clavate versus acuminate), body size (490–720 versus 360–400) and femora III (11 versus 10 chaetae); differ from *P. peruensis* by Th I dorsal chaetotaxy (4 + 4 versus 5 + 5), Abd II ventral (1 + 1 versus 3 + 3) and axial chaetae formula (44/44443 versus 44/44462). For other morphological comparisons between *Paraxenylla* species recorded in Brazil, see Table 7.

**Figure 18:**
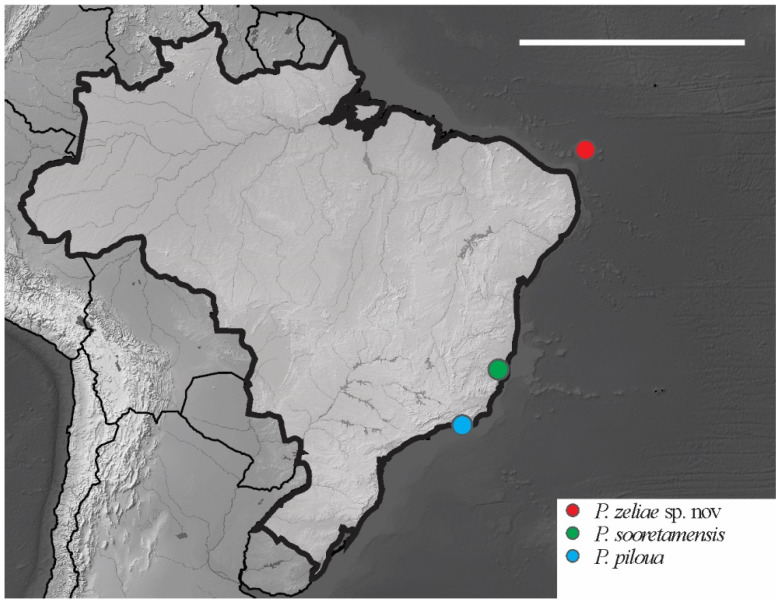
Distribution map for *Paraxenylla* Murphy, 1965 species recorded in Brazil. Scale bar: 3000 km.

*Distribution*. The new species was found on Atol das Rocas Island in the SF sites. The specimen was collected in sandy soil near the psammophile vegetation and seabird guano on rocks. For the congeners registered in Brazil, see Figure 18.

##### Identification Key for *Paraxenylla* Murphy, 1965 Species Recorded in Brazil

1 Ventral tube 1 + 1 chaetae.................................................................................................... 2

– Ventral tube 4 + 4 chaetae.......................... *P. sooretamensis* Queiroz and Deharveng, 2008

2 tenent hair clavate, femora III with 11 chaetae................................. *P. zeliae* sp. nov.

– tenent hair acuminate, femora III with 10 chaetae. *P. piloua* Thibaud and Weiner, 1997

##### *Willemia insularum* sp. nov. Lima and Zeppelini

*Type material*. Holotype female on the slide, Brazil, Rio Grande do Norte, Atol das Rocas, (03°51′50″ S, 33°48′48″ W), intertidal sand, 08–18 September 2015, E.C.A. Lima leg., deposited at the CRFS-UEPB # 12261. Paratypes: 2 males and 3 females on slides, same data as holotype, deposited at the CRFS-UEPB # 12262, 12263, 12264, 12265, 12266. Additional material: 2 females and 1 male on slides, Brazil, Pernambuco, Fernando de Noronha (3°50′58.48″ S; 32°26′3.83″ O and 3°50′36.76″ S; 32°25′12.64″ O), 25–26 July 2012, E.C.A. Lima and A.S. Ferreira leg., deposited at the CRFS-UEPB # 14562, 14563, 14564.

*Description.* Habitus typical of the genus. Color in ethanol white. Tegumental granulation fine and regular. Body length: 490 µm (*n* = 5); holotype measurements as in Table 2.

Body with short acuminate ordinary chaetae, some slightly longer. Sensory chaetae are lanceolate and longer than ordinary chaetae. Antennae are somewhat shorter than cephalic diagonal.

**Figure 19:**
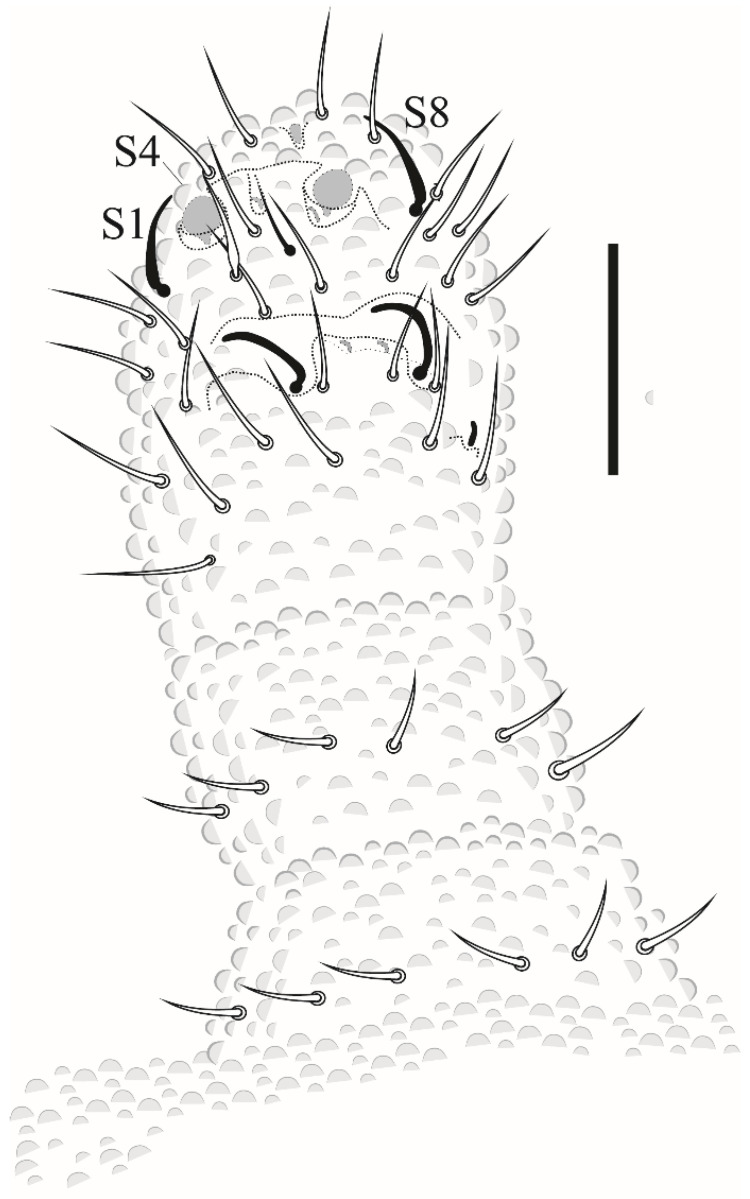
*Willemia insularum* sp. nov. 33, dorsal view of antenna. Scale bar: 10 µm.

Antenna (Figure 19). Ant IV with 4 (S1, S2, S8, S9) subcylindrical sensilla, 2 globular sensilla S7 and S4, hidden each in a separate cavity. Microsensillum (ms) set in the cavity of S7 sensillum. Sensilla S2 and S9 hardly differentiated from ordinary chaetae, S1 and S8 subcylindrical. Apical bulb small and oval, subapical organelle set in a deep cavity, roundish. Sensorial organ of Ant III with 2 long guard chaetae, 2 long subcylindrical sensilla, 1 ventral microsensillum, and a large integumentary fold hiding 2 internal sensilla. Ant II and I with 11 and 6 chaetae, respectively.

**Figure 20:**
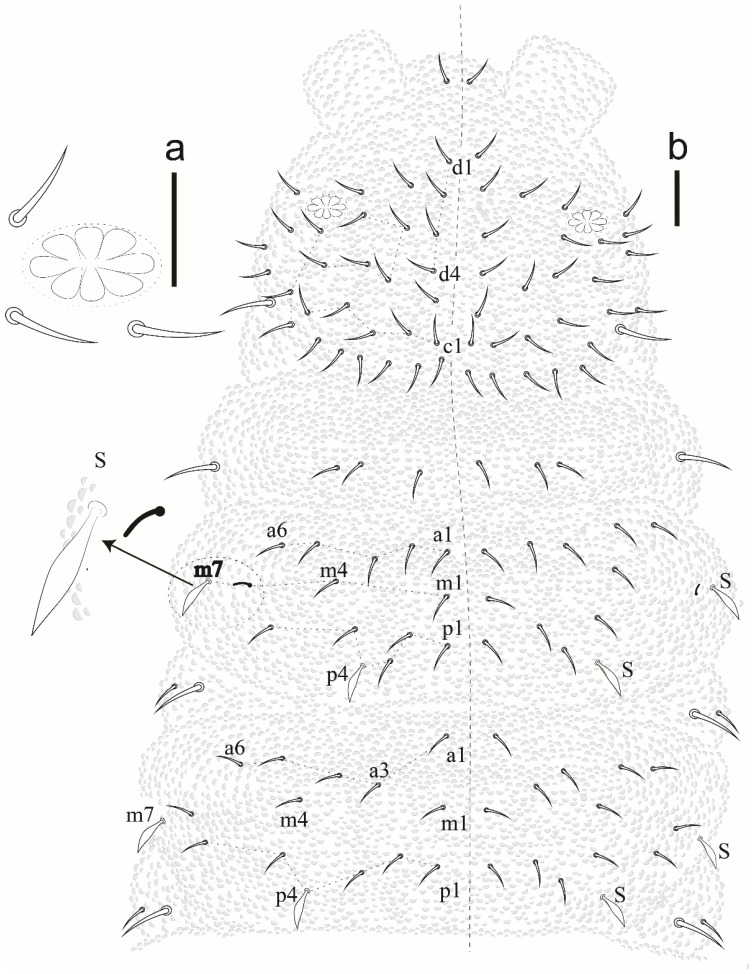
*Willemia insularum* sp. nov. (**a**) postantennal organ, scale bar: 20 µm. (**b**) dorsal view of head and thorax I–III. Scale bar: 10 µm.

**Figure 21:**
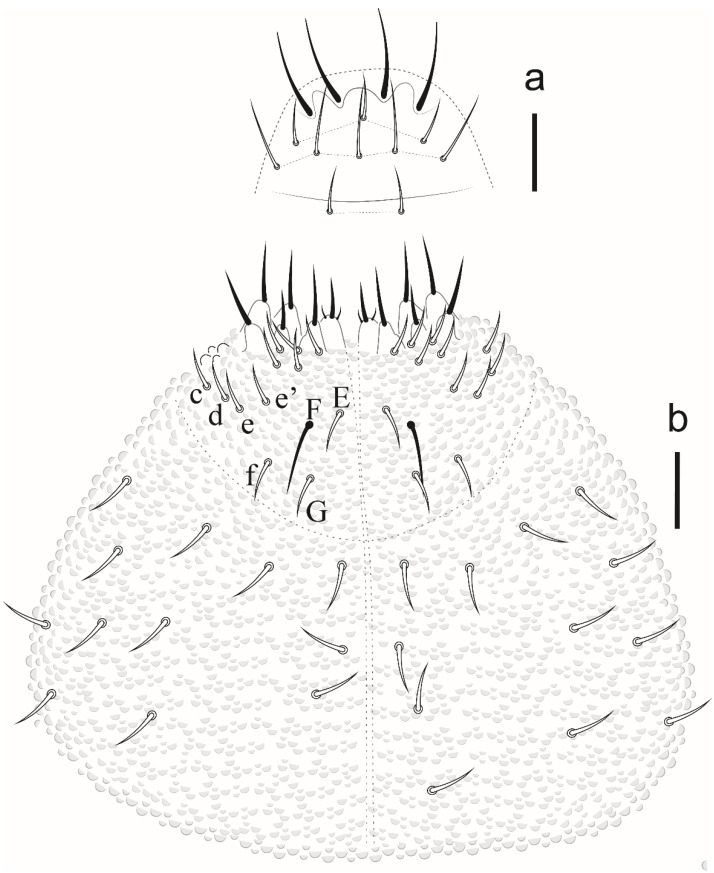
*Willemia insularum* sp. nov. (**a**) prelabrum/labrum, scale bar: 10 µm. (**b**) ventral view of head, scale bar: 10 µm.

Eyes and a0 chaetae absent, PAO with 7–8 vesicles (Figure 20a). Head dorsal (Figure 20b). Prelabrum/labrum with 2/534 chaetae (Figure 21a). Along cephalic groove 3 + 3 chaetae (Figure 21b).

**Figure 22:**
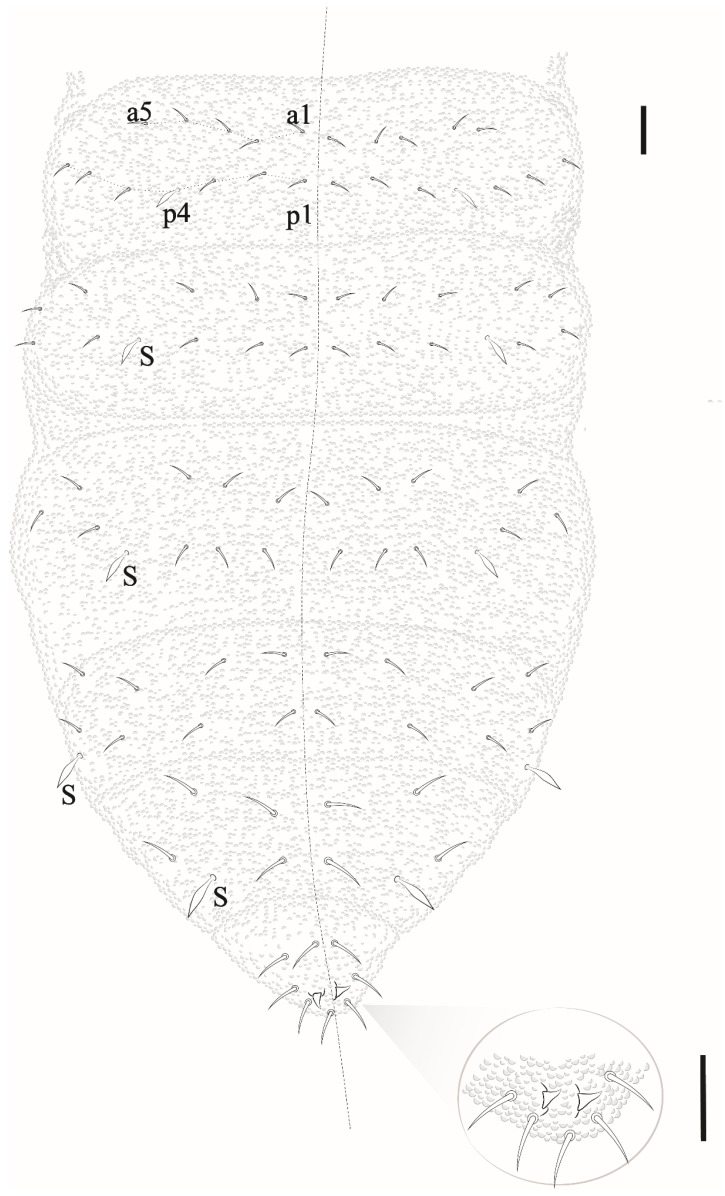
*Willemia insularum* sp. nov., dorsal view of Abd I–VI, anal spine details. Scale bars: 10 µm and anal spine detail, 20 µm.

Dorsal chaetotaxy (Figure 20b and Figure 22). S-chaetae per half tergum formula: 22/11111, in m7 and p4 position on Th II and III, in p4 position on abdominal I to IV and p2 position on Abd V. Sensilla p4 on Th II–Abd I easily differentiated from ordinary chaetae. Abd IV without m-row. With two anal spines on m1 position. 

**Figure 23:**
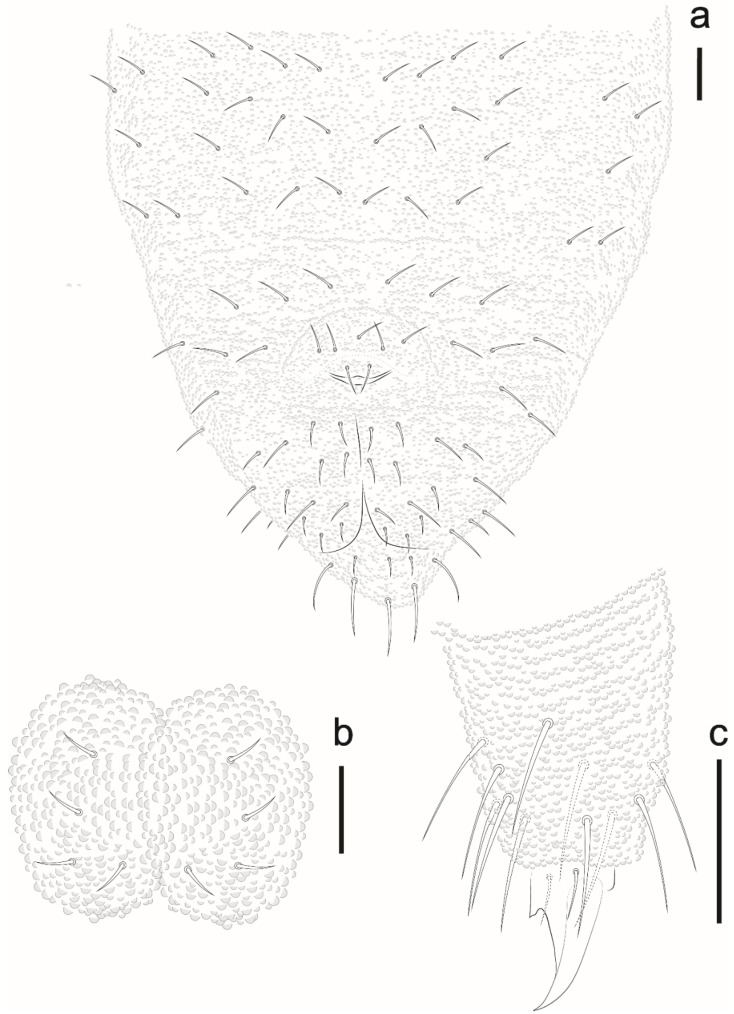
*Willemia insularum* sp. nov. (**a**) ventral view of Abd II–VI, scale bar: 10 µm. (**b**) ventral tubes, scale bar: 20 µm. (**c**) Tita III, scale bar: 20 µm.

Ventral abdominal chaetotaxy is presented in Figure 23a. Sternum of Abd IV with 12 + 12 chaetae. Sternum of Abd II with a-row with 2 chaetae on each side (a3 present). Anal lobes with 16 + 16 chaetae (2 i, 3 hr, e and b absent). Ventral tube with 4 + 4 chaetae (Figure 23b). Tita I, II and III with 12, 12, 11 chaetae. Unguis without teeth, empodial appendage vestigial (Figure 23c).

*Etymology*. An allusion for being the only new species of this study that occurs in two different island regions.

*Remarks*. *Willemia insularum* sp. nov. shares characteristics with the *buddenbrocki*-group species sensu D’Hese 2000 [23,24,25] by S-chaetae S4 and S7 of Ant IV both large, globular, placed in the cavity, and covered in part by tegumental fold and absence of a0 chaetae on the head. The new species presents apical vesicle, Abd V with S-chaetae in p2 position; 3 + 3 chaetae on Th I, presence of anal spines; Abd IV and V with S-chaetae lanceolate. The new species is similar to *W. dhesei* Bu, Potapov and Gao, 2012 (Shandong, Pacific coast of China), *W. delamarei* Prabhoo, 1971 (Indian region); *W. antennomonstrum* Bu, Potapov and Gao, 2012 (Hainan, South China). *Willemia insularum* sp. nov. differs from *W. dhesei* by chaetotaxy of Abd IV (without m-row versus m1 to m4 present) and postantennal organ (7–8 versus 5–6 vesicles). *W. delamarei* has a postantennal organ with 8–12 vesicles, S-chaetae absent in Th II and Abd I while the new species present postantennal organ with 7–8 vesicles and 2 S-chaetae in Th II (m7 and p4) and 1 in Abd I (p4). *Willemia insularum* sp. nov., can be easily distinguished from *W. antennomonstrum* by the sensory organ of Ant III usual for the genus versus 2 expanded and granulated guard sensilla. For other morphological comparisons between *Willemia* species recorded in Brazil, see Table 8.

**Figure 24:**
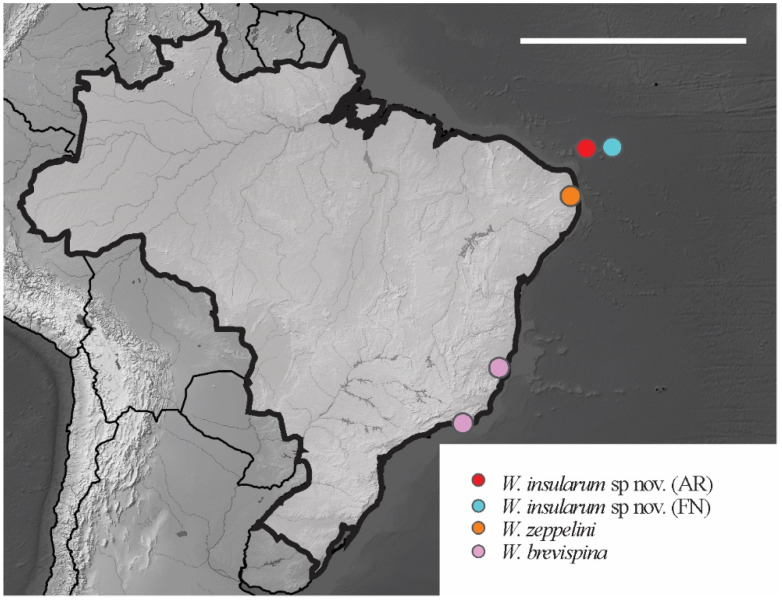
Distribution map for *Willemia* Börner, 1901 species recorded in Brazil. Scale bar: 3000 km.

*Distribution*. The new species were found on Fernando de Noronha and Atol das Rocas Islands in the SF sites. The specimens were collected in sand near the psammophile vegetation, litter and seabird guano. For the congeners registered in Brazil, see Figure 24.

##### Identification Key for *Willemia* Species Recorded in Brazil

1 Anal spine absent……………………………... *W. zeppelinii* D’Hese and Thibaud, 2011

– Anal spine present……………………………………………………………………….. 2

2 Abd IV and V with S-chaetae subcylindrical and acuminate……...…………………. ………………………………………………………………….*W. brevispina* Hüther, 1962

– Abd IV and V with S-chaetae lanceolate…………………….... *W. insularum* sp. nov.

##### Xenylla yucatana Mills, 1938

*Brazilian Oceanic Island Records*. FN (3°50′43.00″ S; 32°25′34.04″ W), SF, 20 Jul 2012, E.C.A Lima and D. Zeppelini leg., CRFS# 14518, 14519, 14522, 14528. FN (3°50′54.21″ S; 32°25′45.56″ W), TF, 20 July 2012, E.C.A Lima and D. Zeppelini leg., CRFS# 14520, 14525, 14527, 14529, 14530, 14531, 14535, 14536, 14553, 14560, 14561. FN (3°50′31.85″ S; 32°24′14.74″ W), TF, 07 August 2012, E.C.A Lima and D.D. Silva leg, CRFS#14474, 14491, 14545, 14546, 14547, 14548, 14549, 14550. FN (3°50′18.66″ S; 32°24′3.09″ W), SF, 07 August 2012, E.C.A Lima and D.D. Silva leg, CRFS# 14479, 14487, 14489, 14492, 14503–14505, 14511–14517, 14521, 14523–14524, 14526, 14532–14537, 14538–14544, 14551–14552, 14555–14556, 14558–14559, 14734, 14735–14768. FN (3°51′3.73″ S; 32°26′0.38″ W), SF, 27 July 2012, E.C.A Lima and A.S. Ferreira leg., CRFS# 14478, 14481, 14484. FN (3°50′36.76″ S; 32°25′12.64″ W), SF, 26 July 2012, E.C.A Lima and A.S. Ferreira leg., CRFS# 9373, 9374–9384, 9423–9533. FN (3°50′48.92″ S; 32°25′13.61″ W), TF, 26 Jul 2012, E.C.A Lima and A.S. Ferreira leg., CRFS# 9532. FN (3°51′50.41″ S; 32°26′5.44″ W), TF, 01 August 2012, E.C.A Lima and A.S. Ferreira leg., CRFS# 14462–14472, 14476–14477, 14480, 14485, 14486, 14493–14502.

**Figure 25:**
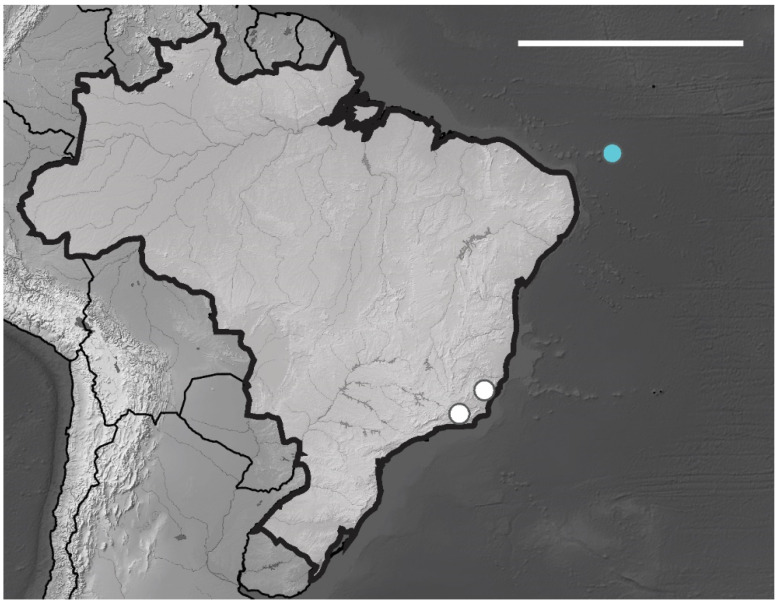
Distribution map for *Xenylla yucatana* Mills, 1938 records in Brazil. Scale bar: 3000 km. Blue circle, Fernando de Noronha; white circles, Brazilian states. Scale bar: 3000 km.

*Brazilian Occurrence.* In Brazil, this species had already been registered in the states of ES, RJ and the island of Fernando Noronha [13] (Figure 25). Cosmopolitan species.

*Biogeographic Distribution.* The type locality of this species in Mexico, Yucatan Peninsula, San Bulha Cenote Motul; specimens collected in bat feces [27]. *Xenylla yucatana* has circumtropical distribution distributed in the following biogeographic regions African Indian Desert (9), East African Steppe (13), South African (14), Madagascar (15), Ascension and St Helena (16), Continental South and East Asia (18), Malaysian (19), Hawaiian (20), New Caledonia (21), Melanesia and Micronesia (22), Caribbean Mainland (24a), Antillean and South Florida (24b), Venezuela and Guyana (25), Northeast and Central Brazilian (27), Andean (28), North and East Australia (32) according to [1].

*Remarks*. The specimens analyzed agree with descriptions by [28,29,30,31]. For other morphological comparisons between *Xenylla* species recorded in Brazil, see Table 9. In the oceanic islands, the species was found in great abundance on the island of Fernando de Noronha in the SF and TF sites.

##### Identification Key for Xenylla Tullberg, 1869 Species Recorded in Brazil

1 With 5 + 5 eyes……………………………………………………………………………… 2

– With 4 + 4 eyes……………………………………………………… *X. yucatana* Mills, 1938

2 Ant IV with 4–5 sensilla………………………………………………………………… 3

– Ant IV with 6 sensilla…………………………………………………………………… 8

3 With 1,1,1 tenent hair…………………………………… *X. brasiliensis* da Gama, 1978

– With 1,2,2 or 2,2,2 tenent hair…………………………………………………………… 4

4 With 1,2,2 tenent hair…………………………………………… *X. welchi* Folsom, 1916

– With 2,2,2 tenent hair……………………………………………………………………… 5

5 Manubrium with 28 chaetae……………………… *X. hodori* Neves and Mendonça, 2017

– Manubrium with more than 28 chaetae………………………………………………… 6

6 Mucro separate from dens……………………………………………………………… 7

– Mucro and dens fused …………………………………………………………………… 9

7 Creased cuticular structure and p6 chaetae present on Abd IV………………………… ……………………………………………………*X. manuelae* Queiroz and Mendonça, 2016

– Creased cuticular structure and p6 chaetae absent on Abd IV………………………………………………………………………………*X. capixaba* Fernandes and Mendonça, 2010

8 With 1,1,1 tenent hair………………………………… *X. nirae* Gama and Oliveira, 1994

– With 1,2,2 tenent hair………………………… *X. wandae* Queiroz and Mendonça, 2016

9 Dens with 2 posterior chaetae……………………………… *X. maritima* Tullberg, 1869

– Dens with 1 posterior chaetae…………………………… *X. subcavernarum* Gama, 1969

#### 3.1.4. Isotogastruridae Thibaud and Najt, 1992

##### *Isotogastrura mucrospatulata* Palacios-Vargas, Lima and Zeppelini, 2013

*Brazilian Oceanic Island Records*. FN (3°50′40.61″ S, 32°25’35.41″ O), SB, 20 July 2012, E.C.A. Lima and A.S. Ferreira Leg., CRFS# 3680–3696. FN (3°50′13.36″ S, 32°24’0.28″ O), SB, 07 August 2012, E.C.A Lima and D. Zeppelini leg., CRFS# 3697–3701. FN (3°50′30.12″ S, 32°25’3.84” O), SB, 20 July 2012, E.C.A. Lima and A.S. Ferreira Leg., CRFS# 3702. FN (3°50′30.12″ S, 32°25′3.84″ O), SB, 20 Jul 2012, E.C.A. Lima and A.S. Ferreira Leg., CRFS# 3703–3708. FN (3°51′19.98″ S, 32°26′43.23″ O), SB, 20 July 2012, E.C.A. Lima and A.S. Ferreira Leg., CRFS# 3709–3710.

*Distribution*: This species is known only in the sand beaches of the Fernando de Noronha Islands. Small body size (350 µm) of *I. mucrospatulata* indicates that it inhabits narrow passages among the grains of sand [32]. Interstitial Collembola looks like typical euedaphic species but is flexible and slender enough to be able to move between sand grains of small size without changing the pore architecture [33]. All specimens in this study were found in the sand at the intertidal zone (SB sites).

*Remark*. The specimens analyzed agree with the original description by [34]. For others, morphological comparisons between *Isotogastrura* species recorded in Brazil, see Table 10.

### 3.2. Species List of Brazilian Poduromorpha

In Brazil, Poduromorpha fauna is registered in 18 states and 3 oceanic islands (Table 11). The states of Acre, Alagoas, Goiás, Maranhão, Roraima, Rio Grande do Sul, Santa Catarina, Tocantins and the oceanic Archipelago of Trindade and Martim Vaz have no record of Poduromorpha. Including the new taxa presented in this study, 139 Brazilian species of Poduromopha distributed in the families Brachystomellidae Stach, 1949 (24 spp.); Hypogastruridae Börner, 1906 (31 spp.); Isotogastruridae Thibaud and Najt, 1992 (2 spp.); Neanuridae Börner, 1901 (71 spp.); Odontellidae Massoud, 1967 (1 sp.); Onychiuridae Lubbock, 1867 (4 spp.); Tullbergiidae Bagnall, 1935 (6 spp.), see Table 2. This number represents an increase of 41 new species records since the last synthesis by [2].

## 4. Discussion

The first Collembola species registered in Brazil was the *Seira musarum* Ridley, 1890 (Entomobryomorpha), whose type locality is Fernando de Noronha. However, after this pioneering study, the only works with Collembola in Brazilian oceanic islands were the description of *Isotogastrura mucrospatulata* Palacios, Lima and Zeppelini 2012 [36] and the species survey done by Lima and Zeppelini, 2015 [3] both in Fernando de Noronha.

Our results show that Poduromorpha fauna of the oceanic islands showed a high percentage of species known only from their type localities and with a great potential for endemisms. All of the Poduromorpha species found in the São Pedro and São Paulo archipelago and Atoll das Rocas are undescribed and with consistent morphological differences in relation to the species found on the continent. Fernando de Noronha, the only Brazilian oceanic island where tourist visitation is allowed, one endemic (*I. mucruspatulata*), four with wide continental distribution and two new species described here. These results may be useful in the management and environmental monitoring programs of oceanic islands in Brazil.

Although the increase in the number of Brazilian Poduromorpha species over the past decade has been relevant (about 35% since [2]) when we consider the variety of Brazilian continental biomes and the complex physiognomy of these areas, current knowledge of the Poduromorpha fauna can still be considered scarce and far from providing a reliable overview of its biogeographical panorama.

## Figures and Tables

**Table 1:** Abbreviations of names of the Brazilian states and oceanic islands. Number of Poduromopha species recorded and region location of each state and oceanic island. Brazilian continental region designations: N—north, NE—northeast, CW—west (central part), SE—southeast, S—south. Brazilian insular region: EA—equatorial Atlantic; SA—South Atlantic. Map shows the geographic position of Brazilian states and oceanic islands.

Abbreviation—State	Species	Region	Abbreviation—Oceanic Island	Species	Region
AC—Acre	0	N	FN—Fernando de Noronha	7	EA
AL—Alagoas	0	NE	AR—Atol das Rocas	2	EA
AM—Amazonas	24	N	SPSP—São Pedro e São Paulo	1	EA
AP—Amapá	2	N	TR—Trindade e Martim Vaz	0	SA
BA—Bahia	1	NE	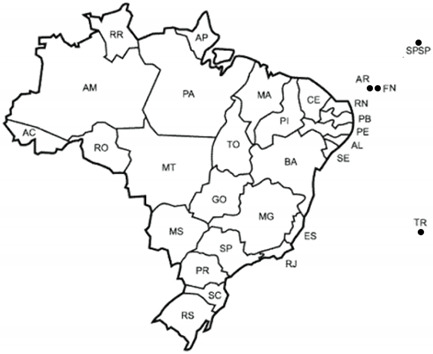		
CE—Ceará	1	NE			
ES—Espiro Santo	17	SE			
GO—Goiás	0	CW			
MA—Maranhão	0	NE			
MG—Minas Gerais	17	SE			
MS—Mato Grosso do Sul	1	CW			
MT—Mato Grosso	5	CW			
PA—Pará	11	N			
PB—Paraíba	4	NE			
PE—Pernambuco	6	NE			
PI—Piauí	2	NE			
PR—Paraná	3	SE			
RJ—Rio de Janeiro	80	SE			
RN—Rio Grande do Norte	5	NE			
RO—Rondônia	1	N			
RR—Roraima	0	N			
RS—Rio Grande do Sul	0	S			
SC—Santa Catarina	0	S			
SE—Sergipe	4	NE			
SP—São Paulo	9	SE			
TO—Tocantins	0	N			

**Table 2:** Holotype measurements for *Friesea noronhaensis* sp. nov., *Friesea rochedoensis* sp. nov., *Willemia insularum* sp. nov., *Acherontiella* sp. and *Paraxenylla zeliae* sp. nov. All measurements in µm.

Character	*F. noronhaensis*	*F. rochedoensis*	*W. insularum*	*Acherontiella.* sp.	*P. zeliae*
Body	528.57	1106.69	520.64	746.69	710.50
Cd	135.52	234.98	78.58	130.36	149.12
Ant I	14.82	33.42	8.93	12.31	14.51
Ant II	16.23	30.08	14.18	16.47	18.75
Ant III	11.97	19.75	16.38	16.38	26,85
Ant IV	19.03	23.07	11.90	31.92	32.63
Mucro	4.52	6.70	Absent	Absent	27.41
Dens	10.41	23.49	Absent	Absent	28.02
Manubrium	22.96	23.87	Absent	Absent	36.28
Unguis III	12.50	20.28	10.57	13.35	18.27
Tita III	22.26	34.61	10.91	18.55	28.40
Femur III	19.82	32.36	17.73	18.09	15.82
Tr	15.55	24.61	9.08	15.50	10.32
Cx	32.53	36.09	12.63	23.92	17.34
Th II ordinary chaetae	4.98	13.16	4.19	7.89	8.02
Th II s-chaetae	11.83	16.17	7.98	13.53	14.22
Anal spine	12.78	20.66	6.97	Absent	Absent
Abd V chaetae a1 or ord.	5.25	19.56	9.65	8.02	8.21
Abd V chaetae p1 or Mac.	13.09	23.10	9.98	8.13	15.03
Abd V s-chaetae	15.08	20.35	21.70	17.08	16.31

**Table 3:** Morphological characters of *Friesea* species record from Brazil. S Abd VI—number of the spine on Abd VI; M Abd VI—modified chaetae on Abd VI; Ten h—tenent hair; BD—body length in μm; AP—apical vesicle; TL—type locality; “?”—missing date; number in Furca column refers of furca “State” according to [12].

Species	Eyes	S Abd VI	Furca	M Abd VI	Ten h	BD	AP	Th I	Tita I-III	TL	Color
*F. arlei* Massoud and Bellinger, 1963	4 + 4	Absent	2	Clavate	1 clavate	?	simple	2 + 2	?	Jamaica	Blue
*F. boitata* Queiroz and Mendonça, 2015	8 + 8	Absent	5	Clavate	4,5,5 clavete	400–800	simple	4 + 4	18,18,17	Brazil	Blue
*F. claviseta* Axelson, 1900	8 + 8	3	2	Clavate	?	550–700				Finland	?
*F. cubensis* Potapov and Banasko, 1985	8 + 8	3	1	Acuminate	Acuminate	670–1000	simple	4 + 4	?	Cuba	?
*F. curupira* Queiroz and Mendonça, 2015	1 + 1	2	5	Acuminate	Acuminate	450–470	simple	4 + 4	15,15,14	Brazil	Dark blue
*F. josei* Palacios-Vargas, 1986	8 + 8	Absent	5	Spinne	3,3,3 clavate	1100	simple	3 + 3	17,17,16	Porto Rico	Blue
*F. jurubatiba* Silveira and Mendonça, 2018	8 + 8	Absent	5	Clavate	4,5,5 clavate	540–680	simple	4 + 4	18,18,17	Brazil	Dark gray
*F. magnicornis* Denis, 1931	8 + 8	3 *	2	Clavate	5 clavate	900	?	?	?	Costa Rica	Gray
*F. mirabilis* (Tullberg, 1871)	8 + 8	3	2	Acuminate	?	?	?	?	?	?	?
*F. noronhaensis* sp. nov.	8 + 8	3	1	Clavate	3,4,4 clavate	440–530	simple	4 + 4	18,18,17	Brazil	Dark blue
*F. reducta* Denis, 1931	8 + 8	Absent	5	Clavate	4,5,5 clavete	600	simple	3 + 3	?	Costa Rica	Gray
*F. rochedoensis* sp. nov.	8 + 8	3	1	Acuminate	3,4,4 clavate	890–1180	simple	4 + 4	18,18,17	Brazil	Light blue
*F. sublimis* Macnamara, 1921	8 + 8	3	2	Clavate	?	?	?	?	?	?	?

* Different sizes.

**Table 4:** Morphological characters of *Arlesia* Handschin, 1942 species recorded from Brazil. BD—body length in mm; AP—apical bulb vesicles; SF—sensory formula; MT—mandibular teeth; TL—type locality.

Species	Eyes	BD	AP	Th I	Tita I-III	Color	SF	MT	TL
*A. albipes* (Folsom, 1927)	5 + 5	0.75–1.5	3	2 + 2	19,19,18	Dark blue	022/11111	4	Panama
*A. arleana* Mendonça and Fernandes, 1999	5 + 5	1.88	3	3 + 3	19,19,18	Black and yellow	022/11111	6	Brazil
*A. intermedia* Fernandes and Mendonça, 2004	5 + 5	0.98–1.6	3	3 + 3	19,19,18	Light gray	022/11111	5	Brazil

**Table 5:** Morphological characters of *Brachystomella* Agren, 1903 species recorded from Brazil.; AP—apical bulb vesicles; PAO— PAO vesicle number; Mx T—maxilla teeth; Ten h—tenent hair; TT— tenaculum teeth; DC—dental chaetae number; TL—type locality; “?” —missing date

Species	Eyes	AP	PAO	Mx T	Th I	Ten h	TT	Tita I–III	DC	TL
*B. septemoculata* Denis, 1931	7 + 7	1	5	8	2 + 2	acuminate	3	19,19,18	5	Costa Rica
*B. garayae* Queiroz and Weiner, 2011	2 + 2	3	5–6	8–9	2 + 2	acuminate	3	19,19,18	5	Brazil
*B. platensis* Najt and Massoud, 1974	8 + 8	2–3	5	7	2 + 2	capitate	3	19,19,18	5	Argentina
*B. parvula* (Schäffer, 1896)	8 + 8	3	6–8	7	3 + 3	capitate	?	19,19,18	5	Germany
*B. agrosa* Wray, 1953	8 + 8	1	4	7–10	2 + 2	acuminate	3	19,19,18	5	Puerto Rico
*B. ceciliae* Fernandes and Mendonça, 2004	8 + 8	1–2	4	8	2 + 2	capitate	3	18,18,17	6	Brazil
*B. nordestina* Souza, Bellini and Weiner, 2018	8 + 8	3	4	6–8	2 + 2	acuminated	3	19,19,18	5	Brazil
*B. villalobosi* Casagnau and Rapoport, 1962	8 + 8	3	3–4	6	3 + 3	capitate	3	18,18,17	3	Brazil
*B. saladaensis* Weiner and Najt, 2001	8 + 8	3	6	7	2 + 2	acuminate	3	19,19,18	5	Argentina
*B. contorta* Denis, 1931	8 + 8	1	5	7	2 + 2	acuminate	3	19,19,18	5	Costa Rica

**Table 6:** Morphological characters of *Acherontiella* Absolon, 1913 species recorded from Brazil. S VI—spine on Abd VI; AL—anterior labral chaetae; Ten h—tenent hair; BD—body length in µm; NS—sensilla number of Ant IV; Tr—trochanter; TL—type locality; “?” —missing date

Species	S VI	AL	Ten h	BD	NS	Th I	Tita I–III	Femur	Tr	TL
*A. globulata* Thibaud e Massoud, 1980	Absent	Smooth	1	520	6	3 + 3	?	?	?	Guadalupe
*A. colotiplana* Palacios-Vargas and Thibaud, 1985	Absent	Smooth	1	450	5	3 + 3	17,17,16	12,11,10	3,4,4	Mexico
*A.* sp. Lima and Zeppelini, 2015	Absent	Spine	1	746	5	3 + 3	18,18,17	11,11,10	3,4,4	Brazil

**Table 7:** Morphological characters of *Paraxenylla* Murphy, 1965 species recorded from Brazil. VT—ventral tube chaetae; TT—tenaculum teeth; VC—ventral chaetae Abd II; Ten h—tenent hair; BD—body length in µm; NS—sensilla number of At. IV; TL—type locality. Ac—acuminate; Cl—clavate.

Species	VT	TT	VC	Ten h	BD	Th I	Tita I-III	Femur	Trochanter	TL
*Paraxenylla piloua* Thibaud and Weiner, 1997	1 + 1	3	1 + 1	4 Ac	360–400	3 + 3	19,19,18	12,12,10	5,5,5	New Caledonia
*Paraxenylla sooretamensis* Queiroz and Deharveng, 2008	4 + 4	3	?	0	440–820	3 + 3	19,19,18	12,12,10	5,5,4	Brazil
*Paraxenylla zeliae* sp. nov.	1 + 1	3	1 + 1	4 Cl	490–720	3 + 3 or 4 + 4	19,19,18	12,12,11	5,5,5	Brazil

**Table 8:** Data matrix of 52 characters coded for Willemia species recorded in Brazil. Character numbers correspond to the characters listed in [26]. Polymorphic characters coded as follow [23], 0 or 1 = a; 0 or 2 = b; 0, 2 or 3 = c. “?”—missing date.

Species	00000 00,000 11111 11,111 22222 22,222 33333 33,333 44444 44,444 55
	01234 56,789 01234 56,789 01234 56,789 01234 56,789 01234 56,789 01
*W. brevispina* Hüther, 1962	13032 03,111 11311 11,101 00122 10,100 01101 11,111 11111 11,111 01
*W. zeppelini* D’Haese and Thibaud, 2011	0c0b2 2b111 01,211 1110? 11,122 10000 01,101 11110 11,001 01111 11
*W. insularum* sp. nov.	13032 03,111 11?11 10,101 00033 11,110 11111 11,111 11011 11,100 01

**Table 9:** Morphological characters of *Xenylla* Tullberg, 1869 species recorded from Brazil.; Ten h—tenent hair; OCM—ordinary chaetae morphology; TL—type locality; MD—mucrodens, MSD—mucro separate from dens; “?”—missing date.

Species	Eyes	Ant IV Sensilla	Furca	Manubrial Chaeta	OCM	Ten h	TL
*X. maritima* Tullberg, 1869	5 + 5	4	MD	?	Smooth	2,2,2	?
*X. nirae* Gama and Oliveira, 1994	5 + 5	6	MSD	?	?	1,1,1	Brazil
*X. brasiliensis* da Gama, 1978	5 + 5	4	MD	?	Smooth	1,1,1	Brazil
*X. subcavernarum* Gama, 1969	5 + 5	4	MD	?	?	2,2,2	Brazil
*X. welchi* Folsom, 1916	5 + 5	4	MSD	34	Smooth	1,2,2	North America
*X. hodori* Neves and Mendonça, 2017	5 + 5	4	MSD	28	Serrated	2,2,2	Brazil
*X. yucatana* Mills, 1938	4 + 4	4	MSD	30	Smooth	2,2,2	Mexican
*X. manuelae* Queiroz and Mendonça, 2016	5 + 5	4	MSD	34	Serrated	2,2,2	Brazil
*X. capixaba* Fernandes and Mendonça, 2010	5 + 5	4–5	MSD	34	Serrated	2,2,2	Brazil
*X. wandae* Queiroz and Mendonça, 2016	5 + 5	6	MSD	34	Serrated	1,2,2	Brazil

**Table 10:** Morphological characters of *Isotogastrura* species record from Brazil modified from [34]; 1, Abd V tegumentary tubercle; 2. Abd I sternite chaetae number; 3. Abd III tergite with a pair of tubercles; 4. Prelabral chaetae (presence vs. absence); 5. Sensillum of Ant III simple or bifid; 6. Dental chaetae; 7. Shape of mucro; 8. Genital chaetae; 9. Number of sensilla on Ant IV; 10. Tubercles on Abd VI; 11. Chaetae on labial triangle; 12. Chaetae on manubrium; 13. Chaetae on furcal subcoxa; 14. Basal manubrium Chaetae. Abbreviations: BI = bifid; H = hooked; MOD = modified; SP = spatulate; un = unmodified; “?”—missing date.

	µm	1	2	3	4	5	6	7	8	9	10	11	12	13	14
*I. mucrospatulata*	350	+	1	—	—	BI	4	SP	MOD	7 + 1	—	4	16	5/2	5 + 5
*I. praiana*	450	+	?	+	+	BI	4	H	un	6	—	5	13	5/2	?

**Table 11:** Species of Poduromorpha recorded from Brazilian states and oceanic islands–updated from [2]. *—New record after [2]. In parentheses, information obtained from non-original reference. “?” following state abbreviation—questionable state record. “un”—unspecified or unknown Brazilian collection habitats. World distribution—see Methods/Species list of Brazilian Poduromorpha. “?” following the distribution abbreviation—questionable distribution record. Habitat—representative type of habitat for the species. “un”—unspecified or unknown habitat (Brazilian distribution, locality abbreviations refer to Brazilian states and Insular locality as listed in Table 1). Family names in bold.

Family/Species	Brazil Distribution	World Distribution	Habitats	Record
**Brachystomellidae Stach, 1949**				
*Brachystomella agrosa* Wray, 1953	FN, PE, BA, PE, RJ, SP	Neo	Halophyte–psammophyte vegetation, sand dunes, flooded areas, pasture, eucalyptus, citric plantation, understory, riparian vegetation, moss, litter and soil over beach rocks, oceanic island	[3,20,35] *
*Brachystomella aspera* (Börner, 1906)	São Francisco	NCB?	un	[36]
*Brachystomella ceciliae* Fernandes and Mendonça, 2004	RJ (ES)	NCB	Halophyte–psammophyte vegetation, litter on sand dunes, flooded areas	[37]
*Brachystomella contorta* Denis, 1931	RJ, (ES)	Bor, Neo, Pal	Halophyte–psammophyte vegetation, sand dunes, beach sand, moss, soil over beach rocks	[37,38]
*Brachystomella garayae* Queiroz and Weiner, 2011	ES	NCB	Forest litter	[39]
*Brachystomella nordestina* Souza, Bellini and Weiner, 2018	RN	NCB	Leaf litter over sandy soil of an urban forested	[40] *
*Brachystomella parvula* (Schäffer, 1896)	RN	Cos	Forest litter	[41]
*Brachystomella platensis* Najt and Massoud, 1974	ES, RJ	Neo, Aus	Beach sand, litter over sandy dunes, halophyte–psammophyte vegetation, guriri vegetation, dunes and supralittoral vegetation	[42,43] *
*Brachystomella saladaensis* Weiner and Najt, 2001	CE	Neo	Leaf litter on the shore of lakes	[44]
*Brachystomella septemoculata* Denis, 1931	RJ	Pal, Neo	Dunes, rotten wood	[45]
*Brachystomella villalobosi* Casagnau and Rapoport, 1962	PE	Neo	un	[35]
*Brachystomellides compositus* Arlé, 1959	RJ	Neo	Beach sand, litter	[46]
*Folsomiella albida* (Arlé, 1959)	RJ	Neo	Leaf litter	[38,46]
*Folsomiella ceca* (Folsom, 1927)	(RJ)	Neo	Soil	[47]
*Folsomiella intermedia* (Arlé, 1939)	RJ	NCB	Soil	[38,48]
*Folsomiella pseudoceca* Mendonça, Fernandes and Abrantes, 2005	RJ	NCB	Soil	[49]
*Folsomiella trisetosa* Mendonça, Fernandes and Abrantes, 2005	RJ, SP	NCB	Soil and rotten logs, soil near streams	[49]
*Maricaella duna* Mendonça and Fernandes, 1997	RJ	NCB	Leaf litter, sand dunes	[50]
*Micronella itacaman* Queiroz and Mendonça, 2013	RJ, MG	NCB	Leaf litter and soil, altitude 2400 m	[51] *
*Micronella longisensilla* Queiroz and Mendonça, 2013	RJ	NCB	Leaf litter and soil, altitude 2100 m	[51] *
*Micronella porcus* (Denis, 1933)	RJ	Neo	Soil	[46]
*Neorganella rotundatae* Queiroz and Mendonça, 2013	RJ, MG	NCB	Leaf litter and soil, altitude 2400 m	[51] *
*Rapoportella pitomboi* Mendonça and Fernandes, 1995	MG, RJ	NCB	Halophyte–psammophyte vegetation, sand dunes, flooded areas	[37,52]
*Setanodosa occidentalis* (Arlé, 1959)	RJ	NCB	un	[38,46]
**Hypogastruridae Börner, 1906**				
*Acherontides eleonorae* Palacios-Vargas and Gnaspini-Netto, 1992	PR, SP	NCB	Guano piles of hematophagous bats in caves	[53]
*Acherontides serrasapoensis* Lima, Stievano and Zeppelini, 2019	MG	NCB	Superficial subterranean habitats and cave	[54] *
*Acherontiella colotipana* Palacios-Vargas and Thibaud, 1985	ES	Pal, Neo	Soil	[55]
*Acherontiella globulata* Thibaud and Massoud, 1980	RJ	Neo	Halophyte–psammophyte vegetation, sand dunes	[37]
*Austrogastrura marambaia* Fernandes, Bellini and Mendonça, 2010	RJ	NCB	Halophyte–psammophyte vegetation	[56]
*Austrogastrura travassosi* (Arlé, 1939)	MS, RJ, PB	Neo	Halophyte–psammophyte vegetation, litter, over rocks and littoral sand	[56,57,58,59]
*Ceratophysella armata* (Nicolet, 1842)	(RJ)	Bor, Cos?, Neo	un	[60]
*Ceratophysella bengtssoni* (Ågren, 1904)	RJ	Bor, NCB	Littoral sand	[59]
*Ceratophysellaengadinensis* (Gisin 1949)	PR, RJ	Neo, Pal	Leaf litter and soil of “campos de altitude”	[61] *
*Ceratophysella nataliae* Cipola, Bellini and Palacios-Vargas, 2017	PR	NCB	Soil, semi-arid vegetation	[62] *
*Ceratophysella rogerarlei* Palacios- Vargas, Bellini and Cipola, 2017	PI	NCB	Soil, anthropized forest from Atlantic forest	[62] *
*Hypogastrura manubrialis* (Tullberg, 1869)	RJ	Cos	un	[63]
*Hypogastrura rehi* Börner, 1906	(SP)	NCB	un	[36]
*Mesogastrura ojcoviensis* (Stach, 1919)	RJ	Bor, Neo	Littoral sand	[59]
*Paraxenylla piloua* Thibaud and Weiner, 1997	RJ	NCB	Halophyte–psammophyte vegetation	[64]
*Paraxenylla sooretamensis* Queiroz and Deharveng, 2008	ES	NCB	Forest litter	[65]
*Paraxenylla zelliae* sp.nov.	AR	NCB	Oceanic islands	sp. nov.*
*Schoetella celiae* Fernandes and Mendonça, 1998	SP	NCB	Forest litter	[66]
*Willemgastrura coeca* Oliveira and Thibaud, 1988	AM, RO	Amz, NCB	Soil	[67]
*Willemia brevispina* Hüther, 1962	RJ, ES	Ant, Neo, Pal	Soil and littoral sand	[55,59]
*Willemia insularum* sp. nov	FN, AR	NCB	Oceanic islands	sp. nov.*
*Willemia zeppelinii* D’Hese and Thibaud, 2011	PB	NCB	Coastal interstitial psammophilous	[23]
*Xenylla brasiliensis* da Gama, 1978	MG, RJ	NCB	Leaf litter, forest soil, foredunes zone, halophyte‒psammophyte vegetation, dunes vegetation and supralittoral vegetation	[43,68] *
*Xenylla capixaba* Fernandes and Mendonça, 2010	ES	NCB	Leaf litter, sand dunes	[42]
*Xenylla hodori* Neves and Mendonça, 2017	AM	Amz	Leaf litter and soil	[69] *
*Xenylla manuelae* Queiroz and Mendonça, 2016	MG, ES, RJ	NCB	Soil leaf litter, altitude 2400 m	[61] *
*Xenylla maritima* Tullberg, 1869	RJ	Cos	Halophyte–psammophyte vegetation, sand dunes, flooded areas	[37]
*Xenylla nirae* Gama and Oliveira, 1994	AM	Amz	Soil	[70]
*Xenylla wandae* Queiroz and Mendonça, 2016	RJ	NCB	Soil and leaf litter of altitude, 2100 m	[61]*
*Xenylla welchi* Folsom, 1916	ES, RJ	Cos	Soil, halophyte–psammophytevegetation, foredunes zone	[55,64]
*Xenylla yucatana* Mills, 1938	FN, ES, RJ	Pal, Neo, Aus	Beach sand, halophyte‒psammophyte vegetation, oceanic islands, dunes vegetation and supralittoral vegetation	[3,42,43] *
**Isotogastruridae Thibaud and Najt, 1992**				
*Isotogastrura mucrospatulata* Palacios-Vargas, Lima and Zeppelini, 2013	FN	NCB	Oceanic islands	[34] *
*Isotogastrura praiana* Silveira, Mendonça and Da-Silva, 2014	RJ	NCB	halophyte‒psammophyte vegetation and dunes vegetation	[71] *
**Neanuridae Börner, 1901**				
*Aethiopella delamarei* Arlé, 1960	MG, RJ, RN	Neo	Leaf litter	[41,46,72]
*Aethiopella littoralis* Fernandes and Mendonça, 2002 *Aethiopella ricardoi* Paz, Bellini and Queiroz, 2019	RJ RN	NCB NCB	Halophyte–psammophyte vegetation, sand dunes, flooded areas sandy soil surrounded by dead foliage near vegetation and lentic freshwater	[73,74] *
*Anurida maritima* (Guérin-Méneville, 1836)	Brazilian coast, PE, SP, RJ, ES, RN	Cos	Surface of rock-pools on the coast	[41,72,75,76,77]
*Arlesia albipes* (Folsom, 1927)	PA, PE, RJ, PB, PI, FN	Neo	Soil, leaf litter, sand dunes and forest, oceanic islands	[38,43,78] *
*Arlesia arleana* Mendonça and Fernandes, 1999	PE	NCB	Leaf litter	[16]
*Arlesia intermedia* Fernandes and Mendonça, 2004	RJ	NCB	Sand dunes, flooded areas	[37]
*Arlesiella amazonica* Arlé, 1966	AM	Amz	Leaf litter	[72,79]
*Australonura gili* Queiroz and Deharveng, 2014	RJ	NCB	Leaf litter and soil of “campos de altitude”	[80] *
*Australanura neotropica* Queiroz and Deharveng, 2014	RJ	NCB	Leaf litter and soil of “campos de altitude”	[80] *
*Brasilimeria anura* Arlé, 1939)	RJ	NCB	Humus under dead leaf	[81]
*Brasilimeria assu* Queiroz and Zeppelini, 2019	RJ	NCB	Leaf litter and soil of Brazilian Páramos	[82] *
*Brasilimeria wygodzinskyi* (Arlé, 1943)	RJ, MG	NCB	un	[83,84]
*Cephalachorutes anneae* Queiroz and Mendonça, 2016	RJ	NCB	Soil leaf litter, altitude 2500 m	[61] *
*Ectonura snowdeni* Queiroz and Deharveng, 2015	MG	NCB	Leaf litter and soil, altitude 2500–2800 m	[85] *
*Friesea arlei* Massoud and Bellinger, 1963	MT	Neo	un	[79]
*Friesea multiclavata* Neves, Mendonça and Queiroz, 2019	AM	NCB	Forest leaf litter of Amazon Rainforest,	[86] *
*Friesea boitata* Queiroz and Mendonça, 2015	RJ	Neo	Soil and leaf litter 2582 m	[87] *
*Friesea claviseta* Axelson, 1900	RJ	Cos	Halophyte–psammophyte vegetation	[64]
*Friesea cubensis* Potapov and Banasko, 1985	RJ	Neo	Littoral sand	[59]
*Friesea curupira* Queiroz and Mendonça, 2015	RJ	NCB	Soil and leaf litter 2500 m	[87] *
*Friesea josei* Palacios- Vargas, 1986	RJ	Neo	Littoral sand	[59]
*Friesea jurubatiba* Silveira and Mendonça, 2018	RJ	NCB	Beach sand and litter of “restinga” soil	[88] *
*Friesea magnicornis* Denis, 1931	RJ	Neo	Halophyte–psammophyte vegetation	[64]
*Friesea mirabilis* (Tullberg, 1871)	RJ	Neo	Halophyte–psammophyte vegetation	[64]
*Friesea noronhaensis* sp. nov.	FN	NCB	Oceanic islands	sp. nov.*
*Friesea reducta* Denis, 1931	RJ	Neo	Neo Halophyte–psammophytevegetation, sand dunes	[37,64]
*Friesea rochedoensis* sp.nov.	SPSP	NCB	Oceanic islands	sp. nov.*
*Friesea sublimis* Macnamara, 1921	ES	Neo, Bor, Pal	Soil	[55]
*Furculanurida belemensis* Arlé and Rufino, 1976	PA	Amz	Soil	[83]
*Furculanurida boiunia* Neves, Mendonça and Queiroz, 2019	AM	Amz	Forest leaf litter of Amazon rainforest	[86] *
*Furculanurida goeldiana* Arlé and Rufino, 1976	PA	Amz	Leaf litter	[83]
*Furculanurida nessimiani* Fernandes and Mendonça, 2002	SP	NCB	Forest litter	[74])
*Furculanurida tropicalia* Queiroz and Fernandes, 2011	ES	NCB	Forest litter	[89]
*Halachorutes schusteri* Arlé, 1966	PA, RJ	Amz, NCB	Leaf litter	[72,77]
*Handschinurida fluminensis* (Arlé, 1939)	RJ, SE	NCB	un	[38,81]
*Handschinurida proxima* (Arlé, 1939)	RJ, SE, SP	NCB	un	[16,38,81]
*Handschinurida rauli* Queiroz and Mendonça, 2014	MG	NCB	Soil and leaf litter, altitude 2700 m	[90] *
*Hylaeanura infima* (Arlé, 1959)	MT, AM, PA, RJ	Neo	Soil, leaf litter, sand dunes, flooded areas	[37,38,46,79]
*Hylaenura mendoncae* Zeppelini and Palacios- Vargas, 2013	MG	NCB	Areas of iron ore mining; leaf litter of semi-deciduous forest fragments	[91]
*Itanura brasiliensis* (Arlé, 1960)	RJ, MG	NCB		[92]
*Kenyura delicata* Arlé, 1966	AM	Amz	Leaf litter	[72,79]
*Kenyura porcula* (Arlé, 1959)	RJ	NCB		[46]
*Kenyura xinguensis* Arlé, 1966	MT	NCB	Leaf litter	[72,79]
*Micranurida fluminensis* Fernandes and Mendonça, 2004	RJ	NCB	Sand dunes, flooded areas	[37])
*Neotropiella arlei* Najt, Thibaud and Weiner, 1990	AM	Neo	Primary and secondary forest soil and litter	[93]
*Neotropiella barbatae* Queiroz, Silveira and Mendonça, 2013	RJ	NCB	Soil and leaf-litter of “campos de altitude” 2400m	[94] *
*Neotropiella carli* (Denis, 1924)	(AM, AP, PA)	Neo	Leaf litter	[38]
*Neotropiella denisi* (Arlé, 1939)	RJ, SE, MT	NCB	Soil, litter on roots	[38,72,81]
*Neotropiella digitomucronata* Thibaud and Massoud, 1983	AM	Neo	Primary and secondary forest soil and litter	[93]
*Neotropiella insularis* Queiroz, Silveira and Mendonça, 2013	RJ	NCB	Leaf litter of Atlantic rainforest	[94] *
*Neotropiella macunaimae* Queiroz, Silveira and Mendonça, 2013	MG	NCB	Soil and leaf litter, altitude 2700 m	[94] *
*Neotropiella meridionalis* (Arlé, 1939)	SE, MT, RJ, MG, PA, AM	Neo	Leaf litter, rotten woods	[38,72,79,81,93]
*Neotropiella minima* Thibaud and Oliveira, 2011	AM	Amz	Floodable rain forest, clay soil	[95]
*Neotropiella plurichaetosa* Thibaud and Oliveira, 2011	AM	Amz	Floodable rain forest, clay soil	[95]
*Neotropiella quinqueoculata* (Denis, 1931)	RJ, AM, AP, MT, PA, RJ	Bor, Neo	un	[35,79]
*Neotropiella silvestrii* (Denis, 1929)	AM	Neo		[95]
*Neotropiella vanderdrifti* Massoud, 1963	AM	Neo	Primary and secondary forest soil and litter	[93]
*Paleonura brasiliensis* (Arlé, 1959)	MG (RJ)	NCB	Leaf litter	[46]
*Paleonura nuda* Cassagnau and Oliveira, 1990	AM	Amz	Primary and secondary forest soil and litter	[96]
*Pronura amazonica* Cassagnau and Oliveira, 1990	AM	Amz	Leaf litter	[96]
*Pseudachorutes bifasciatus* Oliveira and Deharveng, 1994	AM	Amz	Soil	[97]
*Pseudachorutes difficilis* Denis, 1931	RJ, MG, ES	Neo	Sand dunes, flooded areas, soil and leaf litter of highlands grasslands	[37,43] *
*Pseudachorutes gilvus* Oliveira and Deharveng, 1994	AM	Amz	Soil	[97]
*Pseudachorutes herberti* Arlé and Rufino, 1976	AM	NCB	Leaf litter	[83]
*Pseudachorutes massoudi* Arlé, 1966	AM	Amz	Leaf litter	[72,79]
*Pseudachorutes parvulus* Börner,1901	PB	Cos	un	[98]
*Pseudachorutes solaris* Silveira and Mendonça, 2018	RJ	NCB	Sand beach and litter of “restinga”	[88] *
*Pseudanurida sawayana* Schuster, 1965	PE, SP, (RJ)	Bor, Neo, Pal	On sand and seaweed on the rocky coast	[76]
*Tijucameria gabrieli* Mendonça and Silveira, 2012	RJ	NCB	Leaf litter and soil between roots on montane vegetation of the Atlantic rainforest	[99] *
*Tijucameria mame* Mendonça and Fernandes, 2005	RJ	NCB	Forest litter	[100]
**Odontellidae Massoud, 1967**				
*Stachia folsomi* (Arlé, 1968)	PA, RJ	Amz, Pal	Soil, dunes vegetation	[43,101] *
**Onychiuridae Lubbock, 1867**				
*Agraphorura fernandae* (Oliveira and Thibaud, 1992)	PA	Amz	Leaf litter	[102]
*Agraphorura mariapetrae* (Thibaud, 1993)	RJ	Neo	Flooded areas	[37]
*Onychiurus cunhai* Arlé, 1970	PA, AM	Neo	Soil	[58,103]
*Protaphorura cryptopyga* (Denis, 1931)	RJ	Neo, Pal	un	[35]
**Tullbergiidae Bagnall, 1935**				
*Fissuraphorura cubanica* Rusek, 1991	RJ, ES	Neo	Soil, halophyte–psammophytevegetation	[59,63]
*Mesaphorura amazonica* Oliveira and Thibaud, 1992	AM, RJ, ES	Amz, NCB	Litter, halophyte–psammophyte vegetation, sand dunes, soil	[37,55,102]
*Mesaphorura iowensis* (Mills, 1932)	SP	Cos	Soil and litter	[104]
*Mesaphorura maricaensis* Fernandes and Mendonça, 2004	RJ	NCB	Sand dunes	[37]
*Mesaphorura yosiii* (Rusek, 1967)	AM, SP, RJ	Cos	Primary and secondary forest soil and litter; halophyte–psammophyte vegetation, sand dunes and flooded areas, soil, litter	[59,102,104]
*Tullbergia minensis* Arlé, 1959	MG	NCB	Litter	[46]

## Data Availability

All the specimens collected and used in this research are deposited in the *Coleção de Referência de Fauna de Solo* at the State University of Paraiba (CRFS-UEPB) under tumble number as cited in the materials and methods.
